# Mesenchymal stromal cells for steroid-refractory biopsy-proven grade III-IV acute Graft-versus-Host Disease with predominant gastrointestinal involvement

**DOI:** 10.3389/fimmu.2025.1600019

**Published:** 2025-05-13

**Authors:** Adomas Bukauskas, Renata Jucaitienė, Mindaugas Stoškus, Vilma Valčeckienė, Greta Bušmaitė, Artūras Slobinas, Linas Davainis, Inga Šlepikienė, Igoris Trociukas, Valdas Pečeliūnas, Laimonas Griškevičius, Andrius Žučenka

**Affiliations:** ^1^ Hematology, Oncology and Transfusion Medicine Center, Vilnius University Hospital Santaros Klinikos, Vilnius, Lithuania; ^2^ Department of Hematology and Oncology, Institute of Clinical Medicine, Faculty of Medicine, Vilnius University, Vilnius, Lithuania

**Keywords:** acute Graft-versus-Host Disease (aGVHD), steroid-refractory acute Graft-versus-Host Disease (SR-aGVHD), bone marrow-derived MSC (BM-MSC), grade III-IV SR aGVHD, gastrointestinal GVHD, mesenchymal stromal cells (MSC), biopsy-proven SR-aGVHD, severe GVHD

## Abstract

**Introduction:**

Steroid-refractory acute Graft-versus-Host Disease (SR-aGVHD) is a potentially fatal complication occurring in approximately 60-70% of severe grade III-IV GVHD cases, with a higher incidence in patients with gastrointestinal (GI) involvement. GI aGVHD is associated with poor prognosis, with a 2-year overall survival (OS) rate of only 25% in patients with stage 3-4 GI involvement. Mesenchymal stromal cells (MSC) have emerged as a promising therapeutic option due to their favorable efficacy and safety profile. However, data on bone marrow (BM)-derived MSC use in biopsy-proven grade III-IV SR-aGVHD with GI involvement, particularly in stage 3-4 cases, remain limited.

**Methods:**

This prospective, observational, single-arm, single-center study assessed the efficacy and safety of BM-derived MSC for treating adult patients with biopsy-proven grade III-IV SR-aGVHD with predominant GI involvement. Early (1^st^-2^nd^) passage BM-derived MSC were administered weekly at a target dose of 1x10^6^ MSC/kg in two regimens: up to three (MSC3) and six doses (MSC6).

**Results:**

Fifty-seven adult patients with biopsy-proven III-IV grade SR-aGVHD (93% with GI involvement) received MSC treatment. The overall response rate (ORR) was 39% and 42% on Days 14 and 28, respectively, with no significant differences between the two MSC groups (Day 28 ORR 38% for MSC3 and 44% for MSC6). In patients with stage 3-4 GI involvement, the ORR was 26% and 36% at the corresponding time points with comparable efficacy between the two MSC groups (Day 28 ORR 31% for MSC3 and 38% for MSC6). Day 14 and Day 28 responders had significantly higher OS compared to non-responders (52% vs. 7%, p=0.000; 54% vs. 5%, p=0.000), with a comparable OS benefit observed in patients with stage 3-4 GI involvement (45% vs. 8%, p=0.005; 42% vs. 6%, p=0.005), respectively. MSC treatment had a favorable safety profile. The one, 5 and 10-year OS rates were 27%, 24%, and 24%, respectively.

**Conclusions:**

The grade III-IV SR-aGVHD patients, including cases with biopsy-proven severe GI involvement, had significantly better clinical outcomes if responses to MSC treatment were observed on Days 14 and 28. Intensified MSC administration schedule has failed to improve the clinical outcomes. MSC studies focusing on aGVHD prevention and (or) first-line treatment in combination with other agents should be considered.

## Introduction

Acute Graft-versus-Host Disease (aGVHD) is a life-threatening complication after allogeneic hematopoietic stem cell transplantation (HSCT), characterized by immune-mediated injury to the skin, gastrointestinal tract, and liver. Gastrointestinal (GI) manifestation is the most challenging aGVHD to treat and is the primary cause of GVHD-related mortality (1), with severe (stage 3-4) GI GVHD resulting in mortality rates of over 75% (2). Steroid-refractory aGVHD (SR-aGVHD) develops in approximately 60–70% of patients with severe GVHD (3, 4) and with a higher incidence in patients with GI involvement (3, 5, 6). SR-aGVHD is associated with poor prognosis, with long-term survival rates of 25-30% and less than 1-2% for grades III and IV, respectively (7).

Diverse therapeutic interventions to manage SR-aGVHD have failed to improve prognosis (8, 9) and the European Society of Blood and Marrow Transplantation recommends to follow local institutional treatment guidelines and include patients in clinical trials when possible (10).

Two decades ago, *Le Blanc* et al. (11) reported the first successful application of bone marrow (BM)-derived mesenchymal stromal cells (MSC) for aGVHD treatment. The published clinical studies are heterogeneous in terms of the source of mesenchymal stromal cells [bone marrow (12–24), adipose tissue (25, 26), umbilical cord (27, 28), decidua stromal cells (29)], growth media supplement [fetal bovine serum (FBS) (12, 19, 20, 23, 25–27, 30–34), human platelet lysate (HPl) (13–18, 22, 24, 35, 36), both (21)], number of passages [early (P1-P2) (13, 16–18, 24, 28, 36), late (>P2) (12, 20, 22, 33, 35), or early and late (15, 19, 21, 23, 29–31, 34). The age of recipients varies across studies, focusing on adults only (12, 13, 15, 16, 21, 25), both adults and children (14, 18–20, 22–24, 26, 29–33, 35), or exclusively on pediatric populations (34, 36, 37)]. The bioreactor facilitates efficient expansion of MSC in a closed system within a reasonable timeframe, thereby markedly reducing labor and space demands. Bioreactors have emerged as valuable instruments for adhering to stringent Good Manufacturing Practice requirements for cell-based products, particularly in small-scale academic facilities.

Although numerous small-scale studies have investigated BM-derived MSC for the management of SR-aGVHD with predominant GI involvement, only a few have specifically reported outcomes in severe (stage 3-4) GI cases (19, 20) as well as long-term (≥ 2 years) follow-up (14, 16, 17). Furthermore, data on MSC treatment outcomes of histologically confirmed aGVHD are limited, as most studies did not require biopsy for treatment initiation. Differentiating aGVHD from other conditions, such as infections, drug reactions, autoimmune disorders, sinusoidal obstruction syndrome, engraftment syndrome, and dermatologic conditions, is challenging due to the overlapping features like skin rashes, gastrointestinal issues, and liver dysfunction, all of which complicate accurate diagnosis.

In this paper, we demonstrated the long-term efficacy and safety of the treatment of grade III-IV biopsy-proven adult SR-aGVHD with BM-derived early (1st-2nd) passage bioreactor-assisted MSC using two different regimens.

## Materials and methods

### Patient eligibility and study design

**Figure 1:**
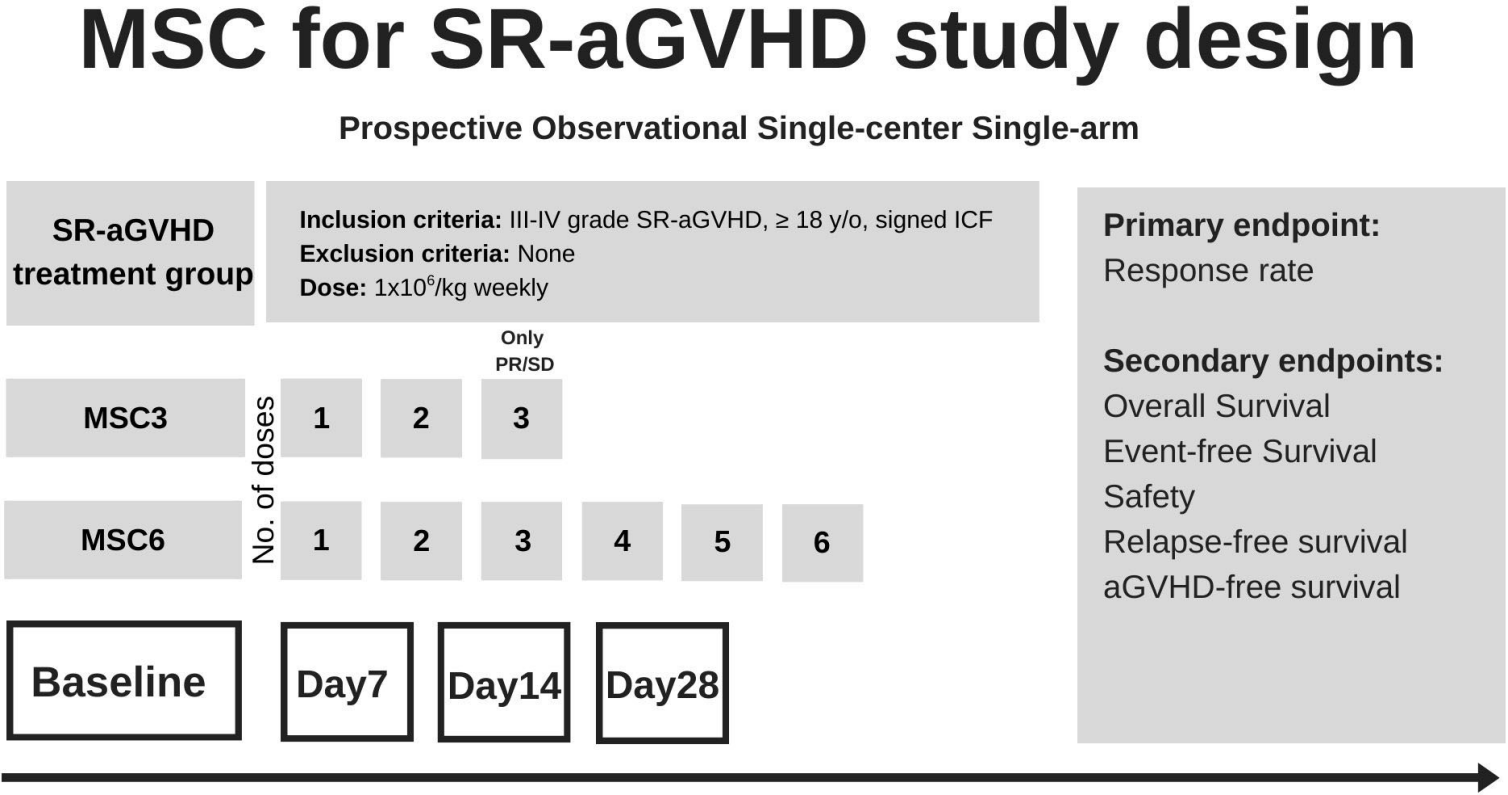
SR-aGVHD mesenchymal stromal cell therapy flowchart and endpoints. ICF, informed consent form; SR-aGVHD, steroid-refractory acute Graft-versus-Host Disease; y/o, years old.

Adult patients with grade III-IV SR-aGVHD (n=57) after allogeneic hematopoietic stem cell transplantation (HSCT) or donor lymphocyte infusion (DLI) at Vilnius University Hospital Santaros Klinikos (Santaros Klinikos) were included. The study was designed to provide real-world data (RWD); thus, no exclusion criteria were applied, except for failure to obtain written informed consent. The patients were included regardless of their performance status, comorbidities, concomitant infections, prognosis, or laboratory findings, which are the exclusion criteria often used in clinical studies. The study flowchart is shown in **Figure 1**.

This was a prospective, observational, single-arm, and single-center study. MSC were offered through a compassionate use program regulated by a special provision of the Lithuanian Health System Law. Histopathological confirmation was mandatory for the diagnosis of aGVHD. The Lerner system (38) and Horn’s adapted criteria from Lerner (39) were used for gastrointestinal and skin histological evaluation, respectively. Staging and grading were based on the modified Glucksberg criteria (40), except for assessing the frequency of diarrhea rather than fecal volume, as proposed by the MAGIC Consortium (1) (**Supplementary Table 1**).

GVHD prophylaxis for matched HSCT included cyclosporine A (CsA) and short courses of methotrexate (Mtx) with mycophenolate mofetil (MMF), substituting CsA in cases of calcineurin-inhibitor toxicity. ATG was added in both matched unrelated and related donor HSCT. In the haploidentical and mismatched donor transplant setting, aGVHD prophylaxis consisted of post-transplant cyclophosphamide, CsA, and MMF.

SR-aGVHD was defined as progression of at least one overall grade within 3 days, failure to demonstrate overall improvement over 5 to 7 days, or incomplete response at 14 days to 1 mg/kg/day methylprednisolone. Budesonide was initiated in all patients upon suspicion of gastrointestinal GVHD. The patients were allowed to continue previously started immunosuppressive therapy.

The study, consisting of two MSC groups, began in October 2013, and the last patient was included in November 2023. The MSC3 and MSC6 groups enrolled patients from October 2013 to December 2015 and from December 2015 to November 2023, respectively. In the MSC3 group, all patients were scheduled to receive two once-weekly MSC doses administered by rapid intravenous infusion. The 3^rd^ dose was reserved for patients who failed to achieve complete response (CR) following two infusions. Following interim analysis of the MSC3 results that demonstrated aGVHD flare-ups after the initial response, the treatment schedule was amended, and patients received six MSC doses administered once weekly by rapid intravenous infusion, regardless of response (MSC6 schedule). The intended weekly MSC dose in both groups was 1 × 10^6^/kg body weight.

This protocol was approved by the Vilnius Regional Bioethics Committee and the study was conducted according to the Declaration of Helsinki. All patients provided written informed consent. MSC were manufactured and administered at Vilnius University Hospital Santaros Klinikos. The study was registered at www.iscrtn.com (#18091201).

### MSC production and administration

MSC production is described in detail in **Supplementary Figure 2**. Briefly, after obtaining informed consent, bone marrow fluid was aspirated from the iliac crest of healthy donors (n=59) under local anesthesia. The medium for MSC expansion was high-glucose DMEM (Invitrogen, Carlsbad, CA) supplemented with 5% human platelet lysate (hPL). BM fluid was processed using a filter-based Bone Marrow MSC Separation Device (Kaneka Corporation, Osaka, Japan) or cultivated without processing. The cells were seeded either in four T-150 flasks or mounted directly on the bioreactor - Quantum Cell Expansion System (Terumo BCT Inc., Lakewood, CO, USA). Four distinct approaches were used to manufacture BM-derived MSC: flask expansion followed by single bioreactor expansion (1F + 1 B), flask expansion followed by two expansions in the bioreactor (1F + 1 B + 2 B), exclusively two bioreactor expansions without flasks (1 B +2 B), and bioreactor expansion from cryopreserved MSC (2 B (cryopreserved)). MSC were harvested, aliquoted into doses of 25, 50, or 75 x 10^6^ cells, cryopreserved using a controlled-rate freezer, and stored in the vapor phase of liquid nitrogen or cryogenic ultralow (-150°C) freezer until release for therapeutic use. The final product was evaluated by flow cytometry for cell-surface molecules CD105, CD90, CD73, CD34, and CD45, viability, and cell count. MSC sterility was assessed by bacterial culture (aerobic, anaerobic, and fungal), mycoplasma DNA, and endotoxin assays. The MSC production flowchart is presented in **Supplementary Figure 2**.

The selected MSC batches were evaluated for differentiation and functional properties (**Supplementary Figure 3**).

Upon request, cryopreserved MSC were immediately (<20 min) delivered to the bone marrow transplantation unit and thawed at the bedside. Cells were administered intravenously via a central venous catheter (CVC) or a peripheral catheter over 5-10 minutes. Each MSC infusion aimed to be 1x10^6^/kg bodyweight. MSC were administered without premedication.

### Study endpoints

The primary endpoint was aGVHD response to MSC therapy. The aGVHD data were recorded prospectively on Days 0, + 7, + 14, +28 after the first MSC infusion. aGVHD was evaluated according to the 1994 Consensus Conference on Acute GVHD Grading Criteria (40). Additional sub-analyses were performed for the D7, D14, and D28 responses.

The secondary endpoints were overall survival (OS), event-free survival (EFS), safety, relapse-free survival (RFS), and aGVHD-free survival (aGVHDFS).

OS was defined as the time from the start of MSC treatment to death from any cause. EFS was defined as the time from the start of the MSC treatment until the following events, whichever occurred first: death, hematologic malignancy relapse, aGVHD relapse after CR/PR requiring next-line treatment, new immunosuppressive agent initiation, and no CR/PR by 3 months of study entry. RFS was defined as the time from the start of MSC treatment to primary disease relapse or death of any cause, whichever occurred first; aGVHDFS was defined as the time from the start of MSC treatment to aGVHD relapse or death of any cause, whichever occurred first. Cyclosporine A, Mycophenolate mofetil, and methylprednisolone dose corrections were not considered the next-line of treatment. The patients not experiencing an event were censored at their last observation. We used the Revised Seattle Classification criteria for organ scoring and the global assessment of chronic GVHD (41).

Data on adverse events of special interest (acute infusion-related reactions, infections requiring treatment, secondary malignancies, and thromboses) were collected.

### Statistical analysis

Comparisons between groups for quantitative data were performed using the independent samples t-test. Spearman rank correlation was used for ordinal variables. Comparisons between groups of categorical data were performed by the chi-square test or Fisher’s precision probability test. The median survival was estimated using the Kaplan-Meier method. The OS, EFS, RFS, and aGVHDFS probabilities were estimated using Kaplan-Meier statistics. The log-rank test assessed the significance of OS, EFS, RFS, and aGVHDFS. Landmark analysis was used to compare survival rates between responders and non-responders. The landmark time points were defined as Days 7, 14, and 28 after the first MSC infusion. The results were expressed as the cumulative incidence probability with a 95% confidence interval. All tests were two-tailed, and a p-value of <0.05 was considered statistically significant. Statistical analyses were performed using SPSS 24 and MS Excel.

## Results

### MSC production

59 healthy donors with a median age of 28 years (range, 20-42), predominantly female (41, 69.5%), donated a median of 25 ml (range, 15-35) of BM for MSC production. A median of 654 × 10^6^ (183-4014) MSC were produced from a single donor. MSC production results are detailed in **Supplementary Table 4**.

### Patient characteristics

**Figure 2:**
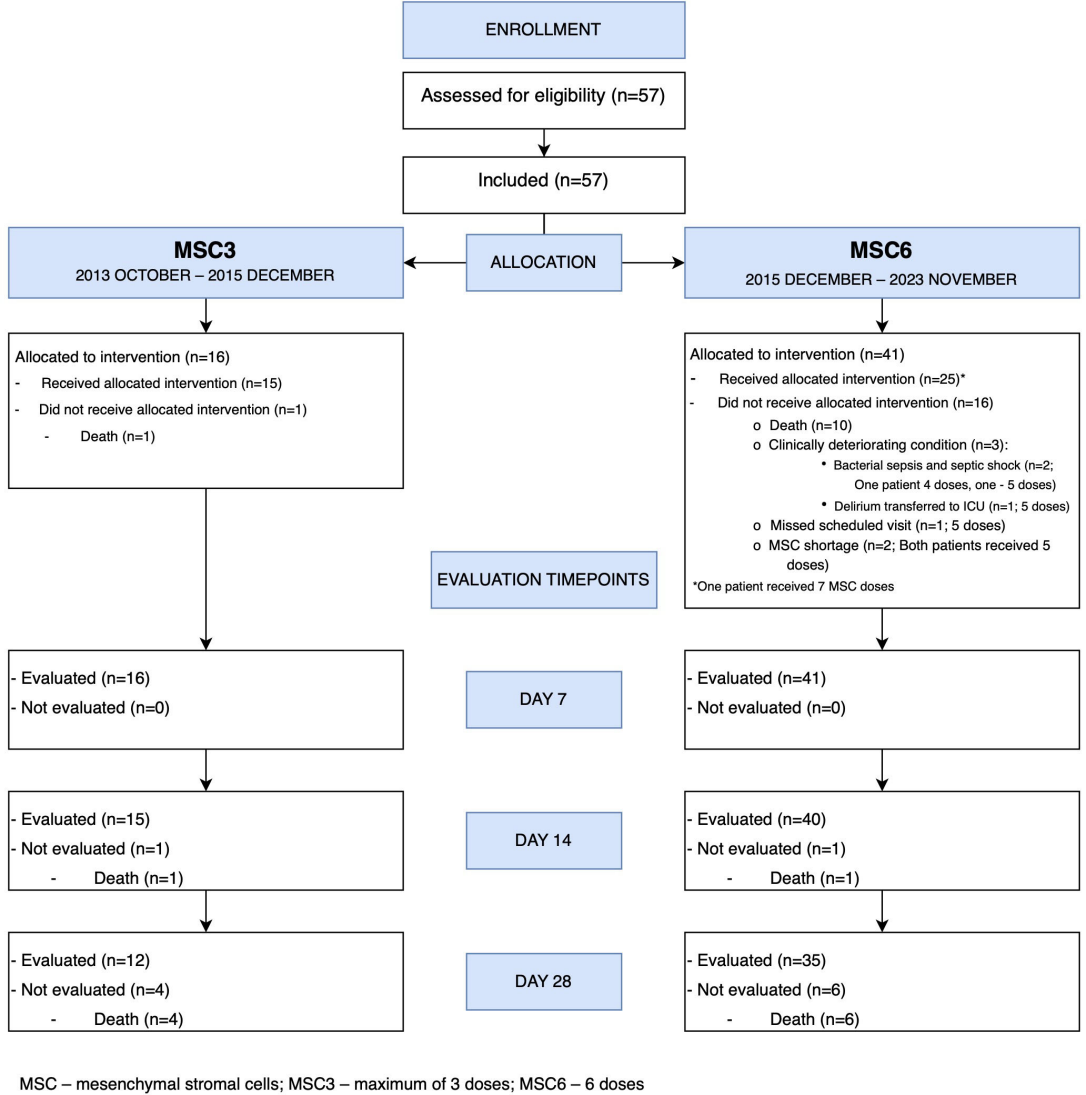
Mesenchymal stromal cells treatment CONSORT flow diagram.

Fifty-seven adult patients, 35 (61%) males and 22 (39%) females, with grades III-IV SR-aGVHD were included (**Figure 2**). Patient characteristics are summarized in **Table 1**.

**Table 1:** Grade III-IV SR aGVHD patient characteristics.

Characteristics	MSC3 (n=16)	MSC6 (n=41)	MSC3+MSC6 (n=57)	MSC3 vs MSC6 *p* value
Patients				
Patient age, years, median (range)	41 (20-60)	58 (19-71)	55 (19-71)	.016
Patient age > 60 years, n (%)	0 (0%)	13 (32%)	13 (23%)	.001
Patient gender, male, n (%)	7 (44%)	28 (68%)	35 (61%)	.130
Primary disease, n (%)				.201
Acute leukemia	9 (56%)	31 (76%)	40 (70%)	
Acute myeloid leukemia, n (%)	5 (31%)	22 (54%)	27 (47%)	
Acute lymphoid leukemia, n (%)	4 (25%)	9 (22%)	13 (23%)	
Other*	7 (44%)	10 (24%)	17 (30%)	
Transplantation				
HSC donor				.969
Matched related donor	6 (38%)	10 (24%)	16 (28%)	
Matched unrelated donor	8 (50%)	23 (56%)	31 (54%)	
Mismatched unrelated donor	2 (12%)	2 (5%)	4 (7%)	
Haploidentical donor	0 (0%)	6 (15%)	6 (10%)	
**HSC donor age, years, median (range)**	37 (22-66)	34 (19-67)	35 (19-67)	.914
**HSC donor gender, male, n (%)**	11 (69%)	31 (76%)	42 (74%)	.341
**Female donors to male recipients**	3 (19%)	7 (17%)	10 (18%)	.884
**Graft source**				.739
PBSCs	16 (100%)	40 (93%)	56 (98%)	
BM	0 (0%)	1 (7%)	1 (2%)	
**CMV status**				.042
Recipient-/Donor-	0 (0%)	3 (7%)	3 (5%)	
Recipient+/Donor-	4 (25%)	18 (44%)	22 (39%)	
Recipient-/Donor+	0 (0%)	0(0%)	0 (0%)	
Recipient+/Donor+	12 (75%)	20 (49%)	32 (56%)	
**Conditioning**				**.046**
MAC	8 (50%)	8 (20%)	16 (28%)	
RIC	8 (50%)	33 (80%)	41 (72%)	
**T-cell depletion**				.168
Pre-transplant ATG	16 (100%)	35 (85%)	51 (89%)	
PT-Cy	0 (0%)	6 (15%)	6 (11%)	
Acute Graft-versus-Host Disease				
**GvHD cause**				1.000
PBSC/BM	14 (88%)	35 (85%)	49 (86%)	
DLI	2 (12%)	6 (15%)	8 (14%)	
**HSCT/DLI to GvHD, days**				.483
Median	44	52	52	
Range	5^#^-158	8-221	5^#^-221	
**GvHD grade**				.613
III	14 (88%)	38 (93%)	52 (91%)	
IV	2 (12%)	3 (7%)	5 (9%)	
Organ involvement				
Skin	4 (25%)	24 (58%)	28 (49%)	.038
Gastrointestinal	15 (94%)	38 (93%)	53 (93%)	1.000
Stage 1-2 Gastrointestinal	2 (12%)	11 (27%)	13 (25%)	
Stage 3-4 Gastrointestinal	13 (81%)	26 (63%)	39 (68%)	
Liver	4 (25%)	10 (24%)	14 (25%)	1.000
**Organ number**				.084
1 organ	9 (56%)	14 (34%)	23 (40%)	
Gastrointestinal	8 (50%)	13 (32%)	21 (37%)	
2 organs	7 (44%)	23 (56%)	30 (53%)	
Skin + Gastrointestinal	3 (19%)	17 (41%)	20 (35%)	
Liver + Gastrointestinal	4 (25%)	4 (10%)	8 (14%)	
Skin + Liver	0 (0%)	2 (5%)	2 (4%)	
3 organs	0 (0%)	4 (10%)	4 (7%)	
Mesenchymal stromal cells				
**MSC treatment line**				.710
2	14 (88%)	32 (78%)	46 (81%)	
>2	2 (12%)	9 (22%)	11 (19%)	
**MSC donor**				.281
Related	1 (6%)	0 (0%)	1 (2%)	
Third-party	15 (94%)	41 (100%)	56 (98%)	
**GvHD onset to 1^st^ infusion, median, days**				.431
Median	15	10	12	
Range	5-126	1-217	1-217	
**MSC dose, x10^6^/kg at day 1**				.116
Median	1.00	0.97	0.97	
Range	0.85-1.33	0.68-1.31	0.68-1.33	
**MSC donors**				**.008**
1	14 (88%)	20 (49%)	34 (60%)	
>1^†^	2 (12%)	21 (51%)	23 (40%)	
**Number of MSC infusions**				**.000**
<=3^‡^	16 (100%)	3 (7%)	19 (33%)	
>3^‡^	0 (0%)	38 (93%)	38 (67%)	

BM, bone marrow; HSC, hematopoietic stem cell transplantation; MAC, myeloablative conditioning; RIC, reduced intensity conditioning; ATG, antithymocyte globulin; GVHD, Graft-versus-Host Disease; PBSC, peripheral blood stem cells; PT-Cy, post-transplant cyclophosphamide; DLI, donor lymphocyte infusion; MSC, mesenchymal stromal cells.

*MSC3 (Chronic lymphocytic leukemia - 1, Chronic myeloid leukemia - 1, Idiopathic myelofibrosis - 1, Myelodysplastic syndrome - 3, Aplastic anemia - 1), MSC6 (Idiopathic myelofibrosis - 2, Diffuse large B cell lymphoma - 1, Multiple myeloma - 1, Myelodysplastic syndrome - 3, Angioimmunoblastic T-cell lymphoma - 3).

#Early GVHD following DLI.

†MSC3 group (2 donors - 1 patient, 3 donors - 1), MSC6 group (2 donors - 19 patients, 3 donors - 2).

‡MSC3 (1 infusion - 1 patient, 2 infusions - 8, 3 infusions - 7) MSC6 (2 infusions - 1 patient, 3 infusions - 2, 4 infusions - 5, 5 infusions - 8, 6 infusions - 24, 7 infusions -1).

The median age of the patients was 55 years (range 19-71). The most common indication for HSCT was acute leukemia (40/57, 70%). Reduced-intensity conditioning was the predominant regimen (41/57, 72%). Except for a single case in which the bone marrow was transplanted, peripheral hematopoietic stem cells were the source of HSCT. Matched unrelated (31/57, 54%) and matched related (16/57, 28%) donors were the most common, followed by haploidentical and mismatched unrelated donors, 6 (10%) and 4 (7%), respectively. In all the cases, aGVHD prophylaxis was administered as scheduled.

### GVHD characteristics

The median time from HSCT or DLI to aGVHD was 52 days (range, 5-221). All patients had severe SR-aGVHD (grades III (52/57, 91%) and IV (5/57, 9%)). The gastrointestinal tract was the most affected organ, accounting for 93% (53/57) of all cases, followed by the skin (49%) and liver (25%). Gastrointestinal and/or skin involvement was confirmed by histopathological evaluation in all the patients, whereas liver involvement was confirmed in 4 of 14 cases. Stage 3-4 GI involvement compromised 39/57 (68%) of all included cases (gastrointestinal histopathological Grade I in 12, Grade II in 12, Grade III in 12, and Grade IV in 3 cases *by Lerner system*). Two organ systems were affected in 30 patients (53%), one organ system in 23 (40%, 21 of 23 (91%) with gastrointestinal involvement), and three organs in 4 (7%).

### MSC therapy

The median time from aGVHD onset to MSC infusion was 15 and 10 days in the MSC3 and MSC6 groups, respectively. MSC were the 2^nd^ line treatment in most SR-aGVHD cases (46, 81%). MSC infusion contained a median of 0.97 x10^6^/kg MSC (range, 0.68 – 1.33 x 10^6^/kg). The median number of infusions was 2 (range, 1-3) and 6 (range, 2-7) in the MSC3 and MSC6 groups, respectively (p=0.000), and neither acute infusion reactions nor thromboses were observed. One patient experienced nausea, and one patient experienced exacerbation of delirium following MSC administration.

### Outcome

The pooled (MSC3 + MSC6) overall response rate (ORR) was 18% [95% CI: 7.50-27.50] (all 10 partial responders (PR)) on Day 7, 39% [95% CI: 25.87-51.73%] (7 complete responders (CR) and 15 PR) on Day 14, and 42% [95% CI: 28.05-56.15%] (15 CR and 9 PR) on day 28. In the SR-aGVHD subgroup of 39 patients with initial stage 3-4 GI involvement, the ORR was 13% [95% CI: 2.22-23.38] (all 5 PR) on day 7, 26% [95% CI: 11.49-39.71] on Day 14 (4 CR and 6 PR), and 36% [95% CI: 17.67-54.13] (9 CR and 5 PR) on Day 28. Regarding GI response in the SR-aGVHD subgroup of patients with initial stage 3-4 GI involvement, the severity of GI GVHD decreased by at least 1 stage in 46% and 54%, by more than 1 stage in 36% and 44%, and completely resolved in 13% and 23% of patients, on Days 14 and 28, respectively.

**Figure 3:**
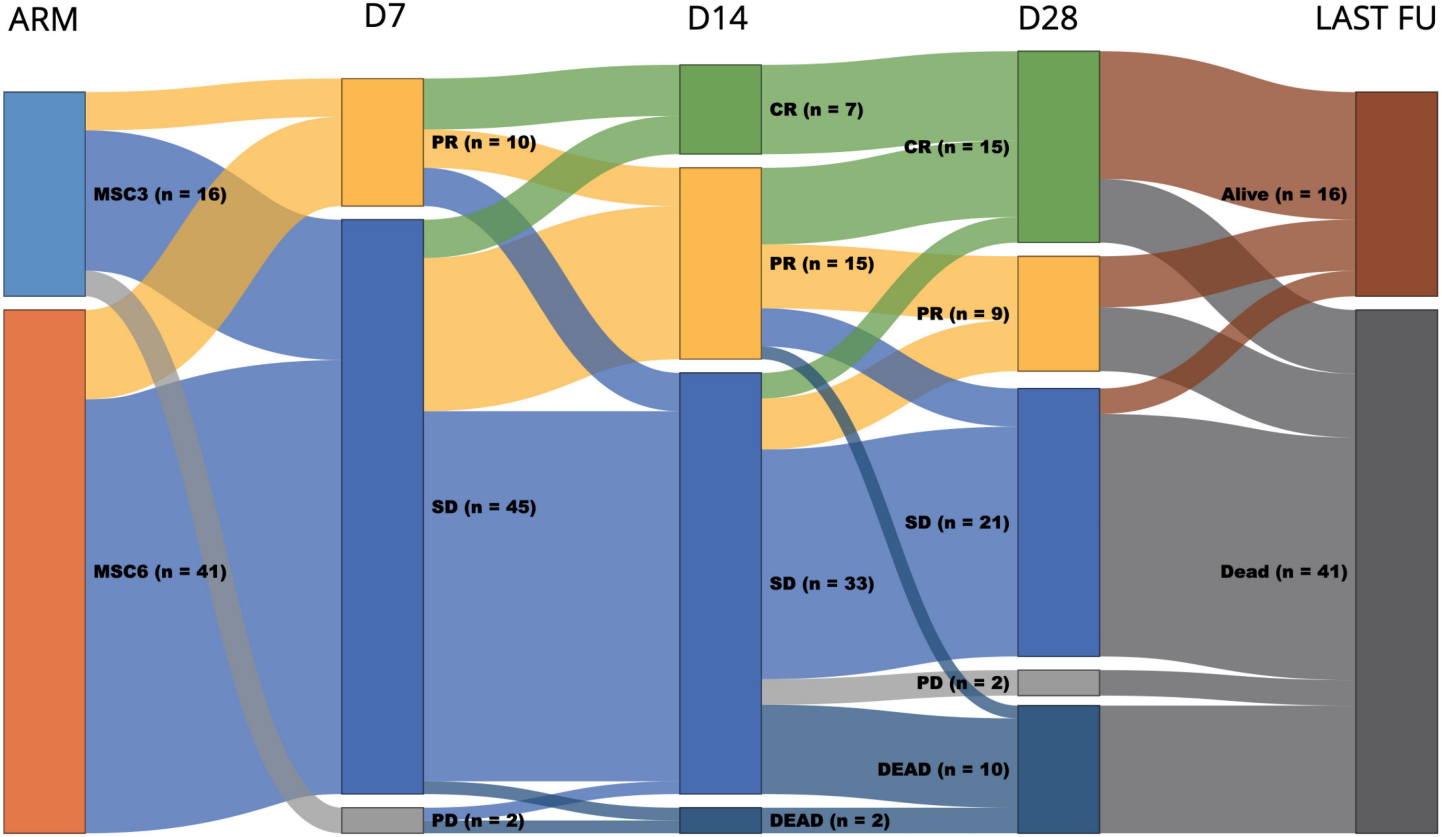
Sunkey diagram of overall response and survival of patients with grade III-IV SR-aGVHD by MSC group. The first column represents the MSC group (MSC3 or MSC6); the second, third, and fourth columns represent treatment response on days 7, 14, and 28, respectively; the fifth column represents the status at the last follow-up; and the width of each bar represents the relative frequency within the cohort. D7, day 7 response; D14, day 14 response; D28, day 28 response; Last FU, last follow-up; CR, complete response; PR, partial response; SD, stable disease; PD, progressive disease.

The MSC treatment response rates are detailed in **Figure 3** and **Supplementary Tables 5–7**.

**Figure 4:**
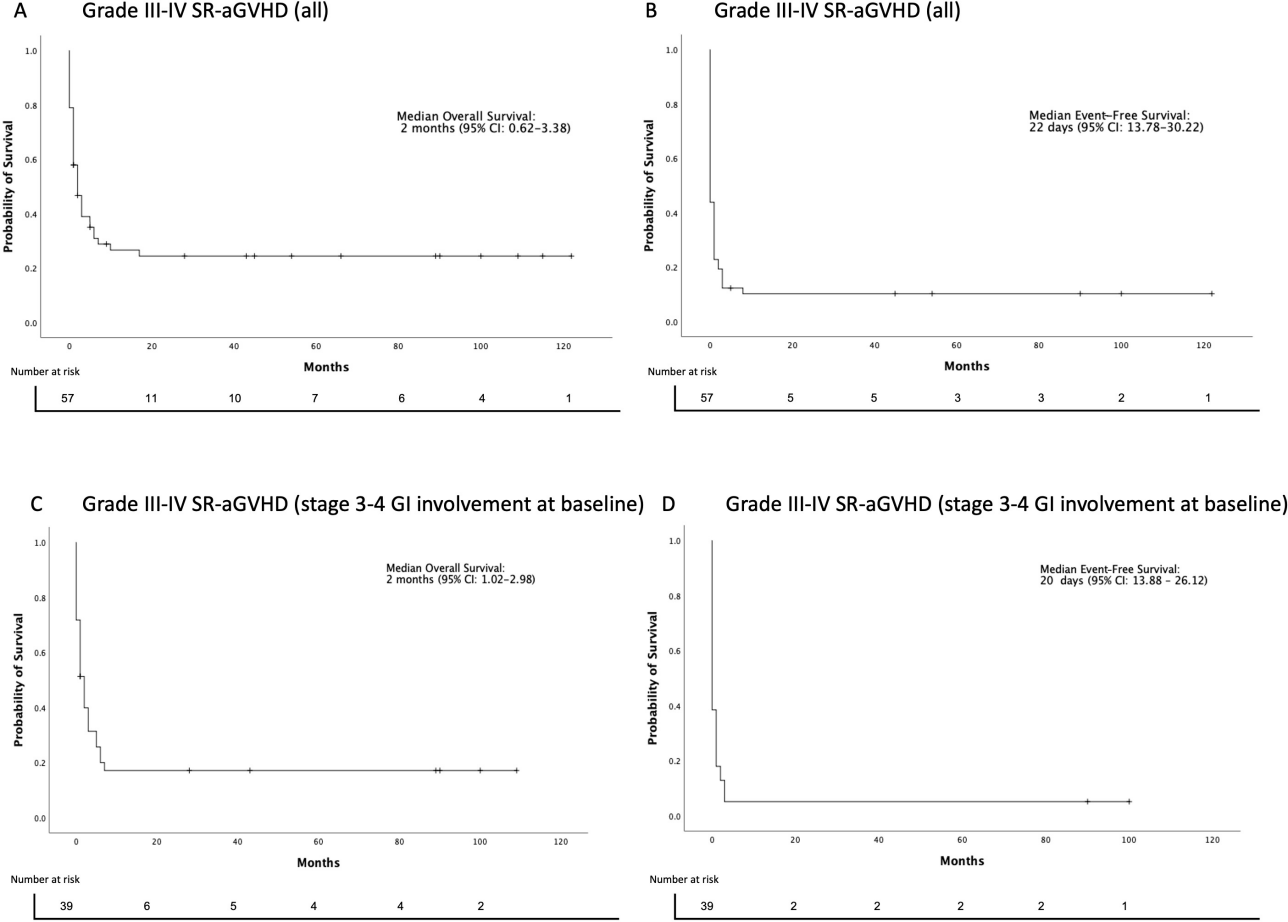
Overall and event-free survival estimates after initiation of MSC treatment. **(A)** Overall survival (OS) and **(B)** Event-free survival (EFS) after MSC initiation (all Grade III-IV SR-aGVHD patients). **(C)** Overall survival (OS) and **(D)** Event-free survival (EFS) after MSC initiation (Grade III-IV SR-aGVHD patients with stage 3-4 GI involvement at baseline).

The data cutoff was December 31, 2023. The median follow-up time of the surviving patients was 44 months (range, 1-122). At the last follow-up, 16 (28%) patients were alive. The median overall survival (OS) was 2 months [95% CI: 0.62-3.38]. The estimated OS at 6 months, 1 year, 5 years, and 10 years was 31% [95% CI: 19–43%], 27% [95% CI: 14–39%], 24% [95% CI: 13-36%], and 24% [95% CI: 13-36%], respectively (**Figure 4A**). The leading causes of death were infection +/- GVHD (31, 75%) and primary disease relapses (8, 20%) (**Table 2**).

**Table 2:** Causes of death in patients treated with MSC.

Causes of death	MSC3	MSC6	Overall
Infection	8 (67%)	11 (38%)	19 (46%)
GvHD and infection	0 (0%)	12 (41%)	12 (29%)
Primary disease relapse	3 (25%)	5 (17%)	8 (20%)
Other*	1 (8%)	1 (4%)	2 (5%)
Total	12 (100%)	29 (100%)	41 (100%)

Data are presented as n (%). *Other: MSC3 – asystole; MSC6 - sudden cardiac death).

**Figure 5:**
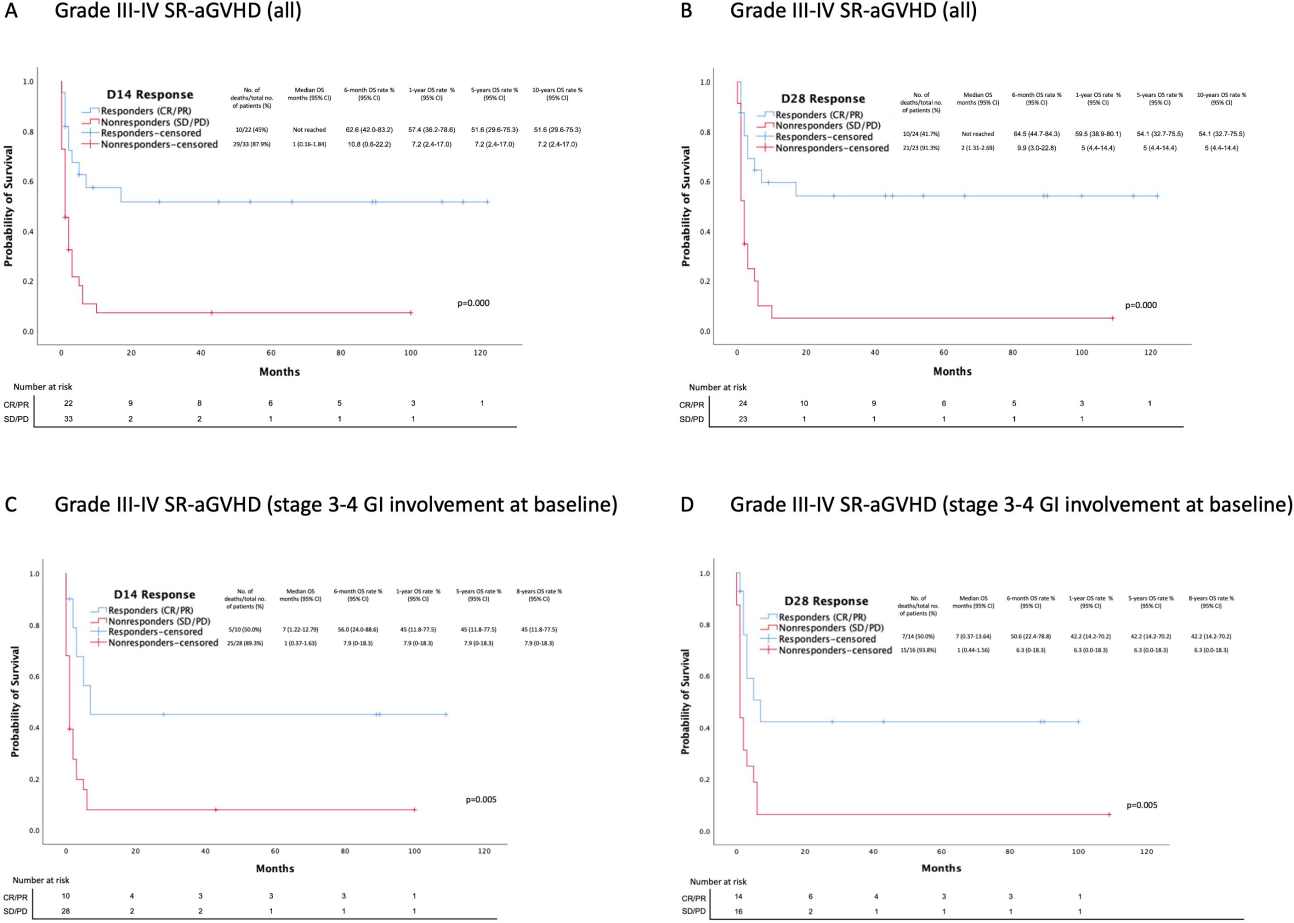
Overall survival estimates after MSC treatment initiation based on D14 and D28 overall response rates. CR, complete response; PR, partial response; SD, stable disease; PD, progressive disease. **(A)** OS between day 14 responders and non-responders (all Grade III-IV SR-aGVHD patients). **(B)** OS between day 28 responders and non-responders. (all Grade III-IV SR-aGVHD patients) **(C)** OS between day 14 responders and non-responders (Grade III-IV SR-aGVHD patients with stage 3-4 gastrointestinal involvement at baseline). **(D)** OS between day 28 responders and non-responders (Grade III-IV SR-aGVHD patients with stage 3-4 gastrointestinal involvement at baseline).

Day 14 and 28 responders had better OS than non-responders, 52% vs. 7% and 54% vs. 5% (p=0.000), respectively (**Figures 5A, B**). In contrast, there was no significant difference in the OS between Day 7 responders and non-responders, 40% vs. 22% (p=0.36). Of 47 non-responders at Day 7, 15 (32%) achieved response at Day 14 and 16 (34%) at Day 28.

In the SR-aGVHD subgroup of patients with stage 3-4 GI involvement, 8 (21%) were alive at the last follow-up. The median OS was 2 months [95% CI: 1.02-2.98]. The estimated OS at 6 months, 1 year, 5 years, and 8 years were 20% [95% CI: 7-33%], 17% [95% CI: 5-29%], 17% [95 CI: 5-29%], and 17% [95% CI: 5-29%], respectively (**Figure 4C**). The leading causes of death were infection +/− GVHD (24, 77%) and primary disease relapse (5, 16%). Similarly, Day 14 and 28 responders at the last follow-up had better OS than non-responders: 45% vs. 8% and 42% vs. 6% (p=0.005), respectively (**Figures 5C, D**). There was no significant difference in OS at the last follow-up between Day 7 responders (n=5) and non-responders (n=34) 0% vs. 20% (p=0.38). Of 34 non-responders at Day 7, 8 (24%) achieved response at Day 14 and 11 (32%) at Day 28.

The median event-free survival (EFS) was 22 days [95% CI: 13.78-30.22] (**Figure 4B**). The most common first event was the next-line of treatment (39, 78%), followed by primary disease relapse (7, 14%). All relapses occurred in acute leukemia patients. No secondary malignancies were observed during 122 months of follow-up. In the SR-aGVHD subgroup of patients with stage 3-4 GI involvement, the median EFS was 20 days [95% CI: 13.88 – 26.12] (**Figure 4D**). The most common first event was the next-line of treatment (27, 73%), followed by primary disease relapse and death (each 5, 13.5%). RFS and aGVHDFS data are presented in **Supplementary 8**.

### MSC3 vs MSC6 groups

Patients in the MSC3 group were significantly younger than in the MSC6 group, with the median ages of 41 years (range, 20-60) and 58 years (19-71), respectively (p=0.016). Thirteen patients in the MSC6 group were over 60 years old. A reduced-intensity conditioning regimen was predominant in the MSC6 group (p=0.046). Skin involvement was more prevalent in the MSC6 group (p=0.038). By design, the MSC6 group patients received more MSC infusions (p=0.000) and from a higher number of different donors (p=0.000) than the MSC3 group (**Table 1**). There were no differences in outcomes or adverse events between the MSC groups (**Table 3**). A similar pattern was observed in the SR-aGVHD subgroup of patients with stage 3-4 GI involvement, with no significant differences in ORR, OS, EFS, aGVHDS, and RFS in both groups (MSC3 – 13 patients; MSC6 - 26 patients).

**Table 3:** MSC3 and MSC6 endpoints.

Endpoint	MSC 3 (n=16)	MSC6 (n=41)	*p value*
ORR (D7)	19% (3 responders – all PR)	17% (7 responders – all PR)	0.884
ORR (D14)	38% (6 responders – 3 CR and 3 PR)	39% (16 responders: 4 CR and 12 PR)	1.000
ORR (D28)	38% (6 responders – all CR)	44% (18 responders: 9 CR and 9 PR)	0.934
OS	2 months (95% CI: 0.55 – 3.95)	2 months (95% CI: 0.37 – 3.63)	0.891
EFS	34 days (95% CI: 12.44 – 55.56)	19 days (95% CI: 8.99 – 29.01)	0.411
aGVHDFS	39 days (95% CI: 27.24 – 50.76)	19 days (95% CI: 7.71 – 30.29)	0.179
RFS	1 month (95% CI: 0.00 – 2.57)	2 months (95% CI: 0.69 – 3.03)	0.878
AEs (during the first 3 months of follow-up)	Febrile neutropenia – 5 Sepsis – 4 CMV reactivation – 5 EBV reactivation – 3 Hypotension – 2 Catheter-related infection, pneumonia, pulmonary failure, ileus, gastrointestinal bleeding, acute cardiac failure – 1	Febrile neutropenia – 6 Sepsis – 18 CMV reactivation – 8 EBV reactivation - 2 ADV infection – 4 Hemorrhagic cystitis (Polyoma JC/BK virus) – 5 Polyoma JC viremia – 1 COVID– 4 Colitis – 2 Ileus – 3 Clostridium difficile– 2 Delirium – 2 Catheter-related infection, influenza virus, acute otitis, blepharoconjunctivitis, renal failure, gastrointestinal bleeding, urinary incontinence, urinary tract parasitic infection, pulmonary candidosis – 1	

ADV, adenovirus; aGVHDFS, acute Graft-versus-Host Disease-free survival; AE, adverse events; CMV, cytomegalovirus; CR, complete response; EFS, event-free survival; EBV, Epstein-Barr virus; ORR, overall response rate; OS, overall survival; PR, partial response; RFS, relapse-free survival.

Of the 23 patients who survived for more than 100 days, nine (39%) developed chronic GVHD (three limited and six extensive) after a median of 291 days (range 108-1133) from the first MSC dose. In patients surviving over 100 days there were three cGVHD (one limited and two extensive) cases within a median of 114 days (range, 108-1133) in the MSC3 group (3 of 7 (43%) and six cGVHD (two limited and four extensive) cases within a median of 328 days (range, 85-957) in the MSC6 group (6 of 16 (38%) (p=0.67). The most commonly cGVHD-affected organs were the skin, gastrointestinal tract, and mouth, 6, 3, and 2, respectively. The remaining cases involved eyes (1), lungs (1), and joints (1).

### Next-line of treatment

**Figure 6:**
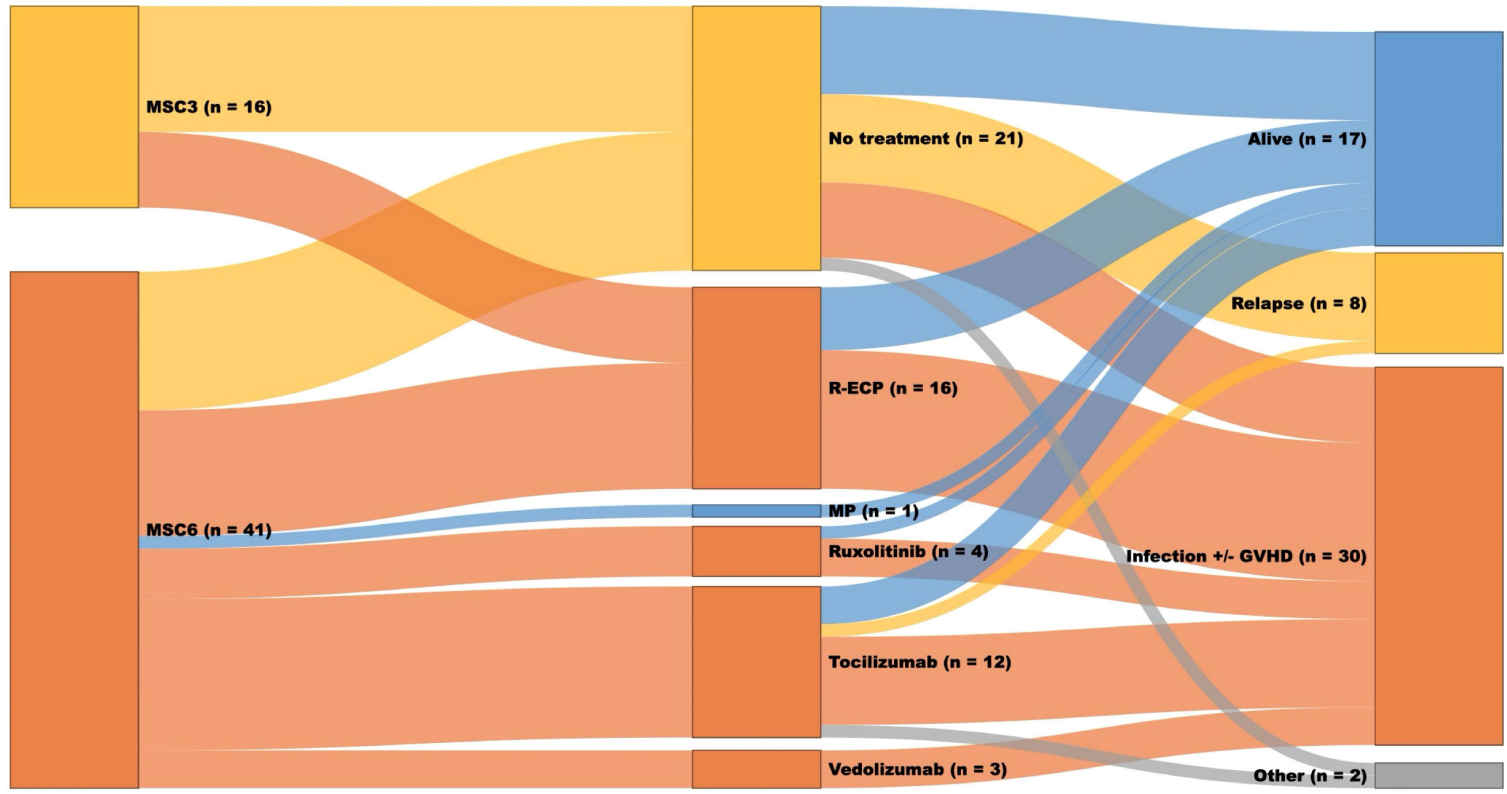
Sunkey diagram of treatment and outcomes in patients with grade III-IV SR-aGVHD receiving the next line of treatment. The first column represents the MSC group (MSC3 or MSC6); the second column represents the next line treatment; the third column represents the status at the last follow-up (alive or dead, if dead, the cause of death: relapse, infection +/- GVHD, other), and the width of each bar represents the relative frequency within the cohort.

The next-line of treatment (**Figure 6**, **Table 4**) was initiated in 36 patients (63%), six (38%) in the MSC3 group, and 30 (73%) in the MSC6 group (p=0.01). The median time from MSC initiation to the 2nd line of treatment was 16 days (range, 0-97), 29.5 days (range, 13-77) in the MSC3 group, and 13 days (range, 0-97) in the MSC6 group (p=0.22). The estimated median OS for patients receiving the next-line of treatment was 2 months [95% CI: 1.11-2.89]. Equally, the estimated median OS for the SR-aGVHD subgroup of patients with stage 3-4 GI involvement receiving the next-line treatment (**Table 5**) was 1 month [95% CI: 0.00-2.09].

**Table 4:** The next-line of treatment after MSC infusion (all patients with grade III-IV SR-aGVHD).

		MSC3 (n=16)	MSC6 (n=41)	MSC3+MSC6 (n=57)	Response (CR/PR)	Dead	Cause of death	Median time to death, (range)
Next-line treatment		6 (38%)	30 (73%)	36 (63%)	12 (33%)	27 (75%)	Infection – 15 Infection + GVHD – 10 Relapse – 1 Other – 1	2 months (0-17) 95% CI: 1.11-2.89
1	R-ECP	6 (100%)	10 (35%)	16 (44%)	6 (38%)	12 (75%)	Infection – 8 Infection + GVHD – 4	2 months (0-7)
2	Vedolizumab	0 (0%)	3 (10%)	3 (8%)	0 (0%)	3 (100%)	Infection – 2 Infection +GVHD – 1	1 month (1-6)
3	Ruxolitinib	0 (0%)	4 (14%)	4 (11%)	1 (25%)	3 (75%)	Infection – 2 Infection + GVHD – 1	1 month (0-2)
4	Tocilizumab	0 (0%)	12 (41%)	12 (33%)	4 (33%)	9 (75%)	Infection – 3 Infection + GVHD – 4 Relapse - 1 Sudden cardiac arrest-1	1 month (0-17)
5	MP rechallenge	0 (0%)	1 (3%)	1 (3%)	1 (100%)	0 (0%)		

R-ECP, Rituximab- Extracorporeal photopheresis; MP, methylprednisolone.

R-ECP median number of procedures – 7 (1-131), MSC3–16 (1-131), MSC6–7 (3-35), Tocilizumab (dose -8mg/kg)– median 3.5 doses (range, 1-8) all in MSC6, Vedolizumab (dose - 300mg) – median 2 doses (range, 2-3) all in MSC6, Ruxolitinib (dose 5-20mg/d) – median 39 treatment days (range, 13-70) all in the MSC6 group.

**Table 5:** The next-line of treatment after MSC infusion (Grade III-IV SR-aGVHD patients with stage 3-4 GI involvement at baseline).

		MSC3 (n=13)	MSC6 (n=26)	MSC3+MSC6 (n=39)	Response (CR/PR)	Dead	Cause of death	Median time to death, (range)
Next-line treatment		6 (46%)	21 (81%)	27 (69%)	9 (33%)	21 (78%)	Infection – 13 Infection + GVHD – 7 Sudden cardiac arrest – 1	1 month (0-6) 95% CI: 0.00-2.09
1	R-ECP	6 (100%)	6 (29%)	12 (44%)	5 (42%)	10 (83%)	Infection – 7 Infection + GVHD – 3	1 month (0-6)
2	Vedolizumab	0 (0%)	3 (14%)	3 (11%)	0 (0%)	3 (100%)	Infection – 2 Infection + GVHD – 1	1 month (1-6)
3	Ruxolitinib	0 (0%)	3 (14%)	3 (11%)	0 (0%)	3 (100%)	Infection – 2 Infection + GVHD – 1	1 month (0-2)
4	Tocilizumab	0 (0%)	8 (38%)	8 (30%)	3 (38%)	5 (63%)	Infection – 2 Infection + GVHD – 2 Sudden cardiac arrest – 1	0 months (0-1)
5	MP rechallenge	0 (0%)	1 (5%)	1 (4%)	1 (100%)	0 (0%)		

R-ECP, Rituximab- Extracorporeal photopheresis; MP, methylprednisolone.

R-ECP median number of procedures – 7 (1-131), MSC3–16 (1-131), MSC6–7 (4-35), Tocilizumab (dose -8mg/kg)– median 5 doses (range, 1-8) all in MSC6, Vedolizumab (dose - 300mg) – median 2 doses (range, 2-3) all in MSC6, Ruxolitinib (dose 5-20mg/d) – median 23 treatment days (range, 13-70) all in the MSC6 group.

The later line treatment is detailed in **Supplementary 9**.

## Discussion

In this paper, we report the long-term real-world data of MSC treatment in 57 adult patients with biopsy-proven grade III-IV SR-aGVHD, of whom 93% and 68% had gastrointestinal (stages 1-4) and severe gastrointestinal (stages 3-4) involvement, respectively. Patients received early (1^st^-2^nd^) passage BM-derived MSC from healthy donors manufactured using a bioreactor-assisted approach. Our findings contribute to the limited knowledge on the most severely affected patient group with grade III-IV SR-aGVHD managed with MSC, particularly in the subgroup with stage 3-4 GI involvement. Additionally, we compared two defined MSC treatment schedules (MSC3 and MSC6 groups) with early and consistent response evaluations. The ORR was 39% and 42% on Days 14 and 28, respectively, with no significant differences between the two MSC groups. However, in the subgroup with severe GI involvement, the ORR was numerically lower at 26% and 36% on Days 14 and 28, respectively. Escalating the treatment intensity from MSC3 to MSC6 did not seem to improve clinical outcomes of grade III-IV SR-aGVHD patients, and those receiving further treatment had a dismal prognosis, with a median survival of only 2 months. The poor 1-year OS of 27% was influenced by the deaths of seven patients due to primary disease relapse without active GVHD. This outcome was largely attributable to the high prevalence of acute leukemia in HSCT recipients, comprising 70% of cases (MSC3 56% and MSC6 76%). However, following the initial drop in 1-year OS, the estimated long-term survival rates at 5 and 10 years remained stable at 24%.

Clinical studies representing adult SR-aGVHD management with BM-derived MSC are summarized in **Supplementary 10**. Response rates vary across studies of adult SR-aGVHD patients treated with MSC, with ORR ranging from 41 to 94% (12–23). Notably, the proportion of less severe grade II patients varied from 27 to 52% in some studies (12, 15, 19, 21). In our study Day 28 ORR was similar to the results (41-54%) of other studies with mostly grade III-IV SR-aGVHD (13, 16, 17, 19). Our 1-year OS was comparable to the 1-year OS rate of 19% (n=11/32) (16), 29% (n=5/18) (17) and 18.2% (n=6/33 (4 children included)) (19) reported in other studies. The causes of death, primarily infections, GVHD, and primary disease relapse, were comparable to those reported in these studies. Beyond smaller-scale studies, several randomized clinical trials have demonstrated favorable outcomes with BM-derived MSC in SR-aGVHD, including combination therapy with basilixamab (42) and single agent MSC (43). The latter led to the U.S. Food and Drug Administration’s approval of remestemcel-L-rknd, an allogeneic BM-derived MSC therapy, for pediatric patients aged 2 months and older.

Our study focused on severe GVHD with GI involvement, as gastrointestinal aGVHD is a well-established risk factor for mortality, with a 2-year OS of only 25% in stage 3-4 GI involvement (2). Notably, 68% of the patients in our cohort fell into this poor prognosis category. There is a lack of comprehensive data in this patient subset, as only a few studies have reported the outcomes of MSC-treated grade III-IV SR-aGVHD patients with stage 3-4 GI involvement, representing 40% and 86% of all the included patients. However, histopathological confirmation of GI involvement was not mandatory in either study (15, 16). Our study demonstrated that in the subgroup of patients with stage 3-4 GI involvement, the ORR was 36% on Day 28 and is in line with an ORR of 44% (50 patients with 3-4 stage GI involvement), reported by *van Dalowsky* et al. (16). Apart from effects on GI, we observed favorable skin SR-aGVHD response to MSC (data in **Supplementary Table 7**), which is consistent with data presented in other publications (13–15, 30) and meta-analysis (44), indicating that, in general, skin responds better to MSC treatment as opposed to visceral SR-aGVHD.

A range of therapeutic strategies with different mechanisms of action have been used for aGVHD (including gastrointestinal manifestation) management, such as extracorporeal photopheresis, anti-thymocyte globulin, antibodies against IL-2Rα and TNF-α, ruxolitinib, vedolizumab, alemtuzumab, fecal microbiota transplantation, tocilizumab, mesenchymal stromal cells, placenta derived decidua stromal cells and other agents are comprehensively summarized in reviews (10, 45–48). Currently, ruxolitinib is the only SR-aGVHD treatment approved by European Medicine Agency (2022). Recent real-world data from 119 adult SR-aGVHD patients (78.2% Grade III–IV and 84% with GI involvement) treated with ruxolitinib demonstrated ORR of 55.9% in Grade III-IV patients and 50% in patients with GI involvement. The 6-month OS was 69.1% in responders and 19.6% in non-responders (49). In ruxolitinib-refractory adults with SR-aGVHD (n=123; 93.5% Grade III–IV), treatment with pooled MSC (MSC-FFM) resulted in Day 28 ORR of 46%, with 1- and 2-year OS rates of 35% and 30%, respectively (50). MSC-FFM were also used in 31 adult patients with predominant severe SR-aGVHD leading to ORR of 77% and 6-month OS of 54% (24). Placenta derived decidua stromal cells (albumin-based formulation) administered to 21 patients, including 18 adults, with biopsy-proven severe GI-aGVHD resulted in Day 28 ORR of 100%, with 1-year and 4-year survival rates of 81% and 57%, respectively (51). Seventy-six SR-aGVHD patients with GI involvement were managed with fecal microbiota - 24 patients in a prospective study (100% with Grade III-IV) and 52 patients in a compassionate use/expanded access program (94% with Grade III-IV) with ORR of 38% and 58%, 1-year OS of 25% and 38%, respectively (52).

Response to MSC is an important predictive factor for long-term outcomes, with Day 28 responses associated with better overall survival in numerous studies (15, 16, 19–21). Unlike previous studies, we evaluated early responses on Day 7 following a single infusion, and on Day 14, after two subsequent weekly infusions, and at universally accepted Day 28 response used to compare results from various studies recommended by PJ Martin et al. (53). In our study, Day 14 and Day 28 responders had significantly better OS than non-responders. In contrast, although OS in Day 7 responders was numerically higher, the small sample size limited statistical significance. Notably, one-third of non-responders at Day 7 achieved response at Day 14 and Day 28. Similarly, in patients with stage 3-4 GI involvement Day 14 and 28 responders had significantly better OS than non-responders whereas no significant difference was observed at Day 7 and no definitive conclusions could be drawn due to small sample size of responders. Nevertheless, one-fourth and one-third of non-responders at Day 7 achieved response at Day 14 and Day 28, respectively. Our study’s ORR on Day 14 was predictive, providing clinically meaningful information for adjusting treatment in non-responders. In our real-world cohort, we could not confirm the findings of Galleu et al. (22), who suggested that response on Day 7 was an early predictor of clinical outcome.

The unmet need for SR-aGVHD patients and their poor prognosis despite numerous available interventions and intensive regimens call for strategies to improve clinical outcomes. Given the extremely poor prognosis of grade III-IV SR-aGVHD in non-responders, aggressive SR-aGVHD treatment with two or more therapeutic interventions should be considered from the outset and not waiting for a response to escalate treatment. Our findings substantiate the safety of MSC therapy, aligning with meta-analyses reporting no major concerns aside from transient fever (54–56). This favorable safety profile supports the potential integration of MSCs into combination treatments or prophylactic management. A recently published randomized phase II clinical trial of 158 patients demonstrated that umbilical cord-derived MSC, administered after haploidentical HSCT, significantly reduced the incidence and severity of chronic and acute Graft-versus-Host Disease (57). However, contradictory findings of GVHD prophylaxis have been reported in meta-analyses: favorable outcomes in preventing aGVHD (44) and little or no difference in the risk of aGVHD (58, 59).

Based on the available data, we suggest that clinical outcomes could be improved by predicting responsiveness to MSC treatment (60–62) and incorporating biomarkers, such as tumorigenicity 2 (ST2) and regenerating islet-derived 3-alpha (REG3α), to predict prognosis. Inclusion of the Day 14 MAGIC algorithm probability biomarker score with the Day 14 Mount Sinai model created three distinct groups (good, intermediate, and poor) with strikingly different non-relapse mortality rates (8%, 35%, and 76%, respectively) (63).

We acknowledge the limitations of this study. It was a single-arm study with variations in concomitant treatment and post-MSC care, and we did not evaluate biomarkers. The MSC used for the treatment did not have identical properties because the patients received therapeutic doses of MSC from different donors, and inter-donor heterogeneity in MSC potency could not be ruled out. We did not investigate the immunosuppressive function and differentiation of every MSC batch, as recommended by ISCT (64). However, the clinical relevance of immunosuppressive function assessment has been criticized (65), and an antagonistic proposal to shift the spotlight from MSC properties to recipient features has also been recommended (60).

In conclusion, grade III-IV SR-aGVHD patients, including cases with biopsy-proven severe GI involvement, had significantly better clinical outcomes if responses to MSC treatment were observed on Days 14 and 28. Intensified MSC administration schedule has failed to improve the clinical outcomes. MSC studies focusing on aGVHD prevention and (or) first-line treatment in combination with other agents should be considered.

## Data Availability

The original contributions presented in the study are included in the article/**Supplementary Material**. Further inquiries can be directed to the corresponding author.
