# Supplementary material for: Mesenchymal stromal cells for steroid-refractory biopsy-proven grade III-IV acute Graft-versus-Host Disease with predominant gastrointestinal involvement
Source: Front Immunol. 2025 May 13;16:1600019. doi: 10.3389/fimmu.2025.1600019 (PMC12106019; doi:10.3389/fimmu.2025.1600019)
Supplement: Supplementary file 1 [file DataSheet1.pdf]

Supplementary Material

Supplement 1. Staging of acute graft-versus-host disease.

Stage	Skin	Liver	Gastrointestinal	Gastrointestinal (adapted by Vilnius University Hospital Santaros Klinikos)
0	No active rash	<35	<500 ml	0-3 times/day, formed stools, analgesics unnecessary for pain management
1	Maculopapular rash <25% of body area	35-50 umol/l	500-1000 ml or persistent nausea, vomiting, or anorexia with a positive upper GI biopsy	4-6 times/day, formed/semi-formed stools, analgesics unnecessary for pain management or persistent nausea, vomiting, or anorexia with a positive upper GI biopsy
2	Maculopapular rash 25 – 50% of body area	51-102 umol/l	1000-1500 ml	7-10 times/day, semi-formed/watery stools, analgesics unnecessary for pain management
3	Generalized erythroderma	103-225 umol/l	>1500 ml	>10 times/day, watery stools, spasmolytics required for pain relief
4	Generalized erythroderma with bullous formation and often with desquamation	>225 umol/l	Severe abdominal pain with and without ileus or grossly bloody diarrhea	>10 times/day, watery stools, opioid required for pain relief or severe abdominal pain with and without ileus or grossly bloody diarrhea

Supplement 2. Summary of MSC production**.**

Volunteer donors underwent medical evaluations to determine their suitability for bone marrow harvest. The evaluation included a medical history, physical examination, and routine blood tests. The donors signed an informed consent form after fully understanding and agreeing to the procedure. The procedure was performed according to the standard practice at VUH Santaros Klinikos. Briefly, the needle was inserted into the posterior iliac crest under local anesthesia in a sterile environment. After bone penetration, 10- or 20-ml sterile plastic syringes containing anticoagulant citrate dextrose solution (ACD-A) were attached to the needle. The ratio of BM fluid to ACD-A was 10:1. The syringes containing the ACD-A and BM fluid mixture were immediately transported to the processing unit at room temperature.

In the processing unit, an additional sample was taken for molecular karyotyping using HumanCytoSNP-12 v2.1 kit (Illumina Inc.). Human BM fluid was either seeded directly without separation or processed through a filter-based Bone Marrow MSC Separation Device (Kaneka Corporation, Osaka, Japan). Cells trapped on the filter were collected in a cell harvesting bag by reverse wash with high-glucose Dulbecco's Modified Eagle Medium (DMEM, ThermoFisher Scientific). The medium for MSC expansion was high-glucose DMEM (Invitrogen, Carlsbad, CA) supplemented with 5% human platelet lysate (hPL). After obtaining consent from the blood donors, human platelets were acquired from the VUH Santaros Klinikos Blood Center. The MSC culture was antibiotic-free. Heparin (5 U/ml) was used to prevent clotting.

MSC were seeded in four T-150 flasks without initial density measurements. The cell medium was exchanged every three days. MSC were harvested after reaching 70-80% confluency. Briefly, the medium was aspirated from the flask and washed with Phosphate Buffer Saline (PBS) (Thermo Fisher Scientific). TrypLE Select (Thermo Fisher Scientific) was used to detach the adherent cells. Afterwards, the medium was added to MSC, which were harvested, counted, and loaded onto the Quantum Cell Expansion System (Terumo BCT, Inc., Lakewood, CO, USA) for passage 1 and, in some instances, subsequent passage 2 expansion. In a few cases, the cells were mounted directly on the bioreactor for Passage 0. The feeding rate and harvest time were guided by lactate and glucose measurements. MSC were harvested, aliquoted into doses of 25, 50, or 75 x 10^6 cells and cryopreserved using a controlled-rate freezer and stored in the vapor phase of liquid nitrogen or cryogenic ultralow (-150° C) freezer until release for therapeutic use. To recapitulate, four distinct approaches were used to manufacture BM-derived MSC: flask expansion followed by single bioreactor expansion (1F + 1 B), flask

expansion followed by two expansions in the bioreactor (1F + 1 B + 2 B), two bioreactor expansions without flasks (1 B + 2 B), and bioreactor expansion from cryopreserved MSC (2 B (cryopreserved)) (Supplement 1 Figure).

The final product was evaluated by flow cytometry for cell surface molecules CD105, CD90, CD73, CD34, and CD45, viability, and cell count. MSC sterility was assessed by bacterial culture (aerobic, anaerobic, and fungal), mycoplasma DNA, and endotoxin testing.

Supplement 2 Figure. Mesenchymal stromal cells production protocol

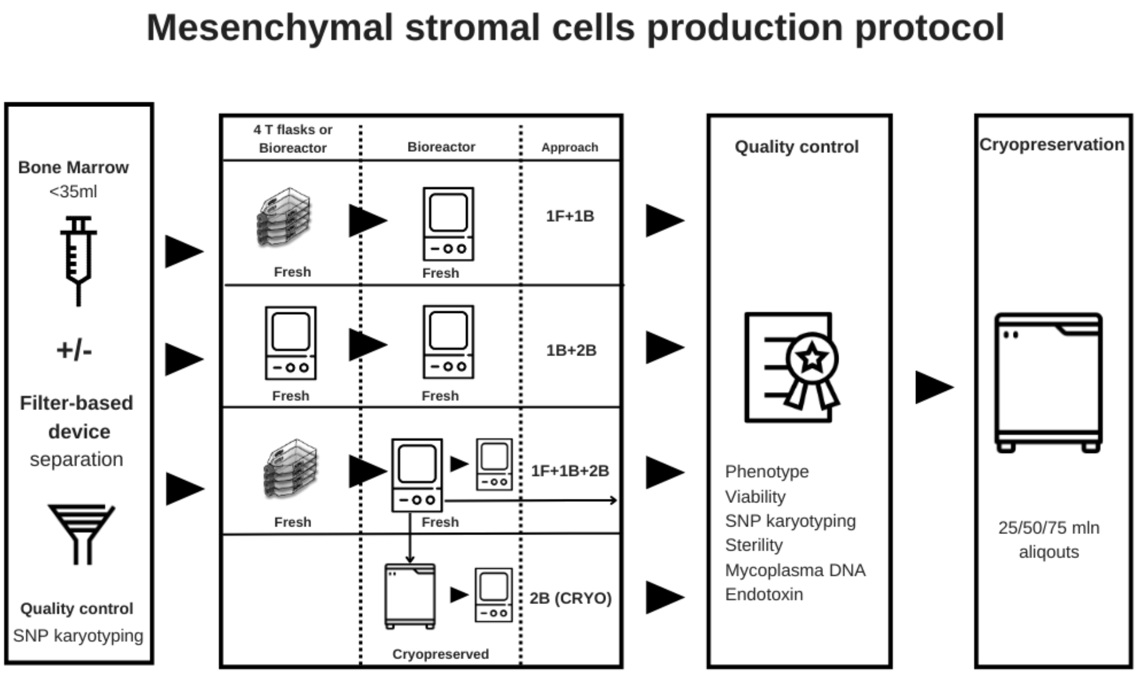

Supplement 3. Assessment of differentiation and function.

The differentiation and immunological properties of MSC were tested *in vitro*. Adipogenic, chondrogenic, and osteogenic differentiation assays were performed using differentiation media and reagents from Sciencell Research Laboratories (Carlsbad, CA) according to the manufacturer's protocols.

IDO expression was analyzed in MSC at both the RNA and protein levels. MSC were treated with 10 ng/ml IFN-γ and 3 ng/ml TNF-α (R&D Systems, Minneapolis, USA) for 24 h to induce IDO production.

For RT-qPCR, RNA was extracted using Trizol (ThermoFisher Scientific (ThermoFisher Scientific) according to the manufacturer's instructions. The expression of IDO-1 and housekeeping genes (GUS, HPRT1, PPIA, PSPMC4, and TBP1) was assayed on a cDNA prepared with RevertAid First Strand cDNA Synthesis Kit and Maxima SYBR Green qPCR Master Mix (both from ThermoFisher Scientific) according to the recommended protocols on a CFX96 system using CFX Manager software (v3.0, Bio-Rad Laboratories, Hercules, CA). The sequences of the forward and reverse IDO-1 primers for qPCR were CTGGGCATCCAGCAGACT and TGAGCTGGTGGCATATATCTTCT, respectively. The sequences of GUS, HPRT1, PPIA, PSPMC4, and TBP1 have been published previously [1].

For immunoblot analysis, cells were washed with PBS and lysed with M-PER reagent according to the manufacturer's recommendations (ThermoFisher Scientific Baltics) for total protein extraction. The IDO protein was determined using anti-IDO antibody (#12006 Cell Signaling Technology, Beverly, MA), and Actin was used as a loading control (#I-19, Santa Cruz Biotechnology, Dallas, TX). Protein extracts were separated on a NuPAGE 4-12% Bis-Tris Gel, transferred on a nitrocellulose membrane, and processed with secondary antibodies using iBind Western System (all from ThermoFisher Scientific).

To determine the effect of MSC on T cell proliferation, MSC were co-cultured with T cells. T cell proliferation was analyzed in response to untreated, and INF-γ and TNF-α treated MSC. MSC were treated with 10 ng/ml IFN-γ and 3 ng/ml TNF-α for 24 h. Human peripheral blood mononuclear cells (PBMC) were extracted by Ficoll gradient separation from healthy donor blood. Isolated PBMC were labeled with CSFE (ThermoFisher Scientific) and plated at $1x10^6$ per well in a 24-well plate. Untreated and treated MSC were added to the T cells at a ratio of 1:10. T cells without MSC were used as a positive control. T cells were stimulated with 0.2 ug/ml anti-human CD3 and CD28 (BD Biosciences). After 4 days of culture, T cell

proliferation was analyzed using *Accuri C6 flow cytometer and CFlow software v1.0 (Accuri Cytometers Inc., Ann Arbor, MI).*

BM-derived MSC from randomly selected three donors were analyzed for differentiation capabilities and immunosuppressive properties. The cells differentiated *in vitro* into three cell types: osteoblasts, chondrocytes, and adipocytes **(Supplement 2. Figure A).** TNF-α and INF-γ induced IDO production in MSC was confirmed at the RNA and protein levels **(Supplement 2. Figure B)** Co-culture with MSC attenuated T cell expansion to 39-48% while pretreatment with INF-γ and TNF-α further suppressed T cell proliferation down to 3-15**% (Supplement 2. Figure C)** confirming *in vitro* the expected immunomodulatory properties of the expanded MSC.

Supplement 3 Figures. MSC differentiation and functional assays.

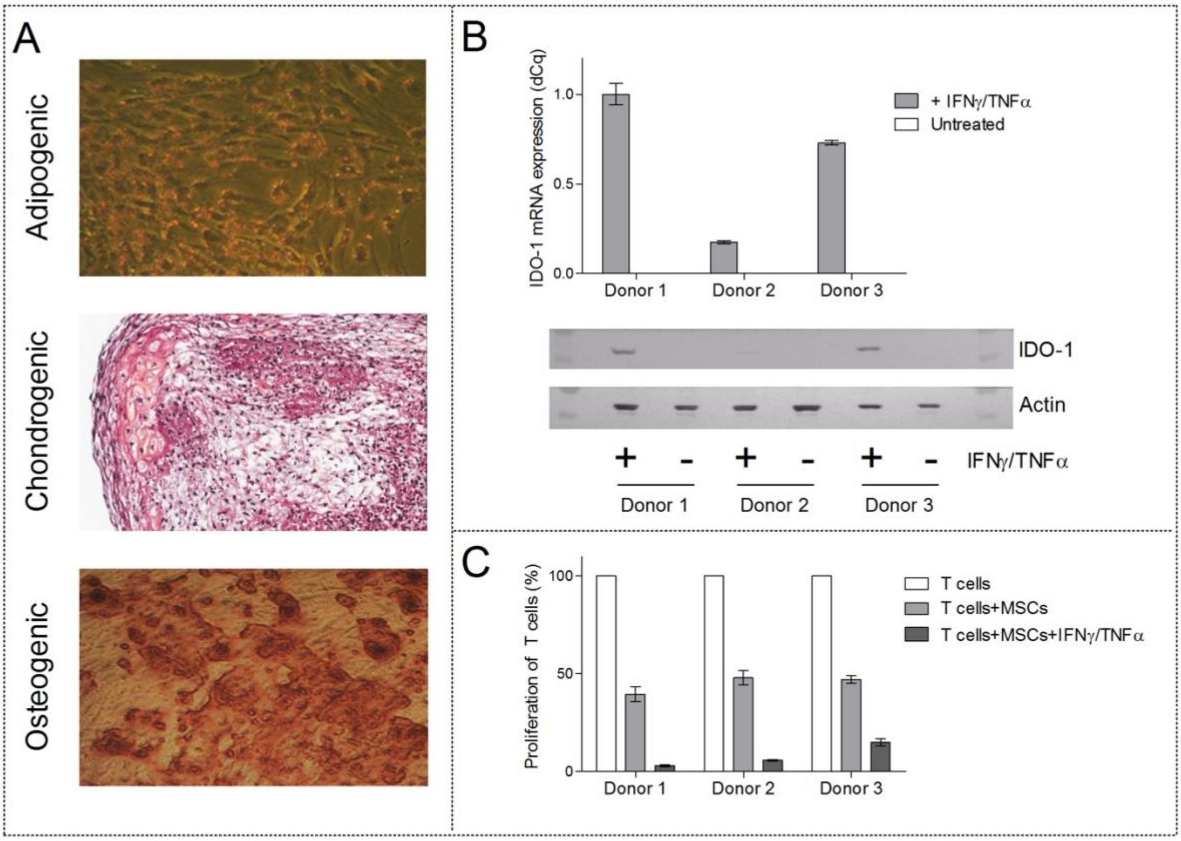

Figure A. Differentiation of MSC into adipogenic, chondrogenic, and osteogenic phenotypes.
Figure B. The expression of IDO-1 at the RNA (upper pane) and protein (lower pane) levels.
Figure C. Proliferation of T cells determined by CSFE proliferation assay with flow cytometry analysis. Data graphed relative to the number of stimulated and proliferating T cells of the respective donor. The experiment was performed in triplicate with MSC derived from three different donors.

Reference:

1. V. Valceckiene, R. Kontenyte, A. Jakubauskas, L. Griskevicius, Selection of reference genes for quantitative polymerase chain reaction studies in purified B cells from B cell chronic lymphocytic leukaemia patients, Br J Haematol 151(3) (2010) 232-8.

Supplement 4. MSC production results.

	1F + 1B (Fresh) (N=26)	1F+1B+2B (Fresh) (N=23)	1B+2B (Fresh) (N=4)	Pooled (Fresh) (N=53)	2B (Cryopreserved) (N=7)
Median age (range)	26 (21-39)	35 (20-42)	24 (21-37)	28 (20-42)	-
Male gender, N (%)	4 (15)	12 (52)	0 (0)	16 (30)	-
Median Initial BM fluid, ml, (range)	25 (15-35)	27 (15-35)	31 (25-35)	25 (15-35)	-
Primary expansion in flasks (P0) duration, days (=1F) (range)	15 (11-29)	14 (13-27)	-	15 (11-29)	-
Primary expansion yield, x10^6 (=1F), (range)	34.75 (0.60-85)	24 (7.50-65)	-	26.1 (0.6-85)	-
Expansion in the bioreactor (P1) duration, days (=1B), (range)	9 (5-38)	11 (8-28)	16.50 (12-20)	11 (5-38)	-
Expansion in the bioreactor yield x10^6 (=1B)	487.50 (183-970)	694 (46-1816)	103.50 (71-138)	547 (46-1816)	-
1B Population doubling time, (range)	58.56 (22.18-179.47)	61.20 (30.76-306.79)	-	59.62 (22.18-306.79)	-
Expansion in the bioreactor (P2) duration days (=2B)	-	10 (7-14)	8.50 (6-14)	10 (6-14)	12 (10-14)
Expansion in the bioreactor yield x10^6 (=2B) (Fresh cells)	-	431 (105 – 1115)	355 (323-1159)	394 (105-1159)	450 (132-855)
2B Population doubling time, hours	-	80.35 (45.65-359.28)	102.14 (46.90-184.54)	83.06 (45.65-359.28)	84.83 (70.56-102.62)
Overall (1B+2B, Fresh) Population doubling time, hours	-	69.34 (51.56-213.96)	-	69.34 (51.56-213.96)	-
Yield, x10^6 (1F+1B)	487.50 (183-970)	694 (46-1816)	-	547 (46-1816)	-
Final yield. x10^6 (1F+1B+2B)	-	1188 (307-2102)	458 (393.50-1297)	635 (183-2102)	-
Time from seeding to yield, days (1F+1B)	25 (17-56)	26 (22-42)	-	26 (17-56)	-
Time from seeding to yield, days (1F+1B+2B)	-	37 (32-52)	25.50 (20-31)	-	-

Abbreviations: BM, bone marrow; F, flask; B, bioreactor; P, passage.
Six (10%) BM MNC cultures failed to expand, and 3(5%) cultures were found to be infected (Staphylococcus epidermidis, Bacillus subtilis, and Staphylococcus Warneri were identified as infecting microorganisms). A median of 654 x 10^6 (183-4014) MSC were produced from a single donor (including batches produced from cryopreserved MSC (2B (Cryopreserved); N=6)). The median viability was 98.80 (range, 87.00-99.90) (evaluated 83/88 samples; 5 not evaluated). ISCT flow cytometric phenotype criteria were met in 98.85% for CD90 (evaluated 87/88 samples), 97.72% for CD105 (evaluated 87/88), 75.86% for CD73 (evaluated 87/88), 98.86% for CD34 (evaluated 87/88), and 100% for CD45 (evaluated 68/88). The evaluated batches were devoid of mycoplasma DNA and endotoxin levels of <5 EU/ml. None of the mesenchymal stromal cells acquired chromosomal aberrations during clinical grade expansion, as determined by SNP karyotyping.

Supplement 5. Response rates at different time points after the first administration of MSC (all Grade III-IV SR-aGVHD patients).

Characteristics	Day 7	Day 14	Day 28
**MSC3 (n=16)**			
**CR**	0 (0%)	3 (18.75%)	6 (37.50%)
**PR**	3 (18.75%)	3 (18.75%)	0 (0%)
**ORR**	**3 (18.75%)**	**6 (37.50%)**	**6 (37.50%)**
**SD**	11 (68.75%)	9 (56.25%)	6 (37.50%)
**PD**	2 (12.50%)	0 (0%)	0 (0%)
**DEAD**	0 (0%)	1 (6.25%)	4 (25.00%)
**MSC6 (n=41)**			
**CR**	0 (0%)	4 (9.75%)	9 (21.95%)
**PR**	7 (17.07%)	12 (29.27%)	9 (21.95%)
**ORR**	**7 (17.07%)**	**16 (39.02%)**	**18 (43.90%)**
**SD**	34 (82.93%)	24 (58.54%)	15 (36.59%)
**PD**	0 (0%)	0 (0%)	2 (4.88%)
**DEAD**	0 (0%)	1 (2.44%)	6 (14.63%)
**MSC3+MSC6 (n=57)**			
**CR**	0 (0%)	7 (12.28%)	15 (26.32%)
**PR**	10 (17.54%)	15 (26.32%)	9 (15.79%)
**ORR**	**10 (17.54%)**	**22 (38.60%)**	**24 (42.11%)**
**SD**	45 (78.95%)	33 (57.89%)	21 (36.84%)
**PD**	2 (3.51%)	0 (0%)	2 (3.51%)
**DEAD**	0 (0%)	2 (3.51%)	10 (17.54%)

Abbreviations: CR, complete response; PR, partial response; ORR, overall response rate (ORR=CR+PR); SD, stable disease; PD, progressive disease.
Data are presented as n (%). Table shows the response rates of 57 adult patients with grade III–IV SR-aGVHD (MSC3 – 16 and MSC6 – 41 patients). Two (3.51%) patients were not evaluated for day 14 response and ten (17.54%) for day 28 response due to early death.

Supplement 6. Response rates at different time points after the first administration of MSC (Grade III-IV SR-aGVHD patients with stage 3-4 gastrointestinal involvement at baseline).

**Characteristics**	**Day 7**	**Day 14**	**Day 28**
**MSC3 (n=13)**			
**CR**	0 (0%)	2 (15.38%)	4 (30.77%)
**PR**	2 (15.38%)	1 (7.69%)	0 (0%)
**ORR**	**2 (15.38%)**	**3 (23.08%)**	**4 (30.77%)**
**SD**	9 (69.23%)	9 (69.23%)	5 (38.46%)
**PD**	2 (15.38%)	0 (0%)	0 (0%)
**DEAD**	0 (0%)	1 (7.69%)	4 (30.77%)
**MSC6 (n=26)**			
**CR**	0 (0%)	2 (7.69%)	5 (19.23%)
**PR**	3 (11.56%)	5 (19.23%)	5 (19.23%)
**ORR**	**3 (11.56%)**	**7 (26.92%)**	**10 (38.46%)**
**SD**	23 (88.46%)	19 (73.08%)	10 (38.46%)
**PD**	0 (0%)	0 (0%)	1 (3.85%)
**DEAD**	0 (0%)	0 (0%)	5 (19.23%)
**MSC3+MSC6 (n=39)**			
**CR**	0 (0%)	4 (10.26%)	9 (23.08%)
**PR**	5 (12.82%)	6 (15.38%)	5 (12.82%)
**ORR**	**5 (12.82%)**	**10 (25.64%)**	**14 (35.90%)**
**SD**	32 (82.05%)	28 (71.80%)	15 (38.46%)
**PD**	2 (5.13%)	0 (0%)	1 (2.56%)
**DEAD**	0 (0%)	1 (2.56%)	9 (23.08%)

Abbreviations: CR, complete response; PR, partial response; ORR, overall response rate (ORR=CR+PR); SD, stable disease; PD, progressive disease.
Data are presented as n (%). The table shows the response rates of 39 adult patients with grade III-IV SR-aGVHD with stage 3-4 gastrointestinal involvement at baseline (MSC3 – 13 and MSC6 – 26 patients). One patient (2.56%) was not evaluated for the day 14 response, and nine (23.08%) for the day 28 response due to early death.

Supplement 7. SR-aGVHD response by organ system.

Pt. No	GROUP	SKIN				GASTROINTESTINAL				LIVER			
		B	D7	D14	D28	B	D7	D14	D28	B	D7	D14	D28
1	MSC3	0	0	0	0	4	4	4	0	0	0	0	0
2	MSC3	4	4	3	0	0	0	0	0	0	0	0	0
3	MSC3	0	0	5	5	4	4	5	5	1	2	5	5
4	MSC3	4	4	4	5	3	4	4	5	0	2	3	5
5	MSC3	0	0	0	0	4	3	3	3	3	2	2	2
6	MSC3	0	0	0	0	2	1	0	0	0	0	0	2
7	MSC3	0	0	0	0	4	4	4	4	0	0	0	0
8	MSC3	0	0	0	0	4	4	4	0	0	0	0	0
9	MSC3	0	0	0	0	4	3	2	2	0	0	0	1
10	MSC3	3	0	0	0	3	2	0	0	0	0	0	0
11	MSC3	0	0	0	0	4	4	4	3	0	0	0	1
12	MSC3	0	0	0	5	2	2	1	5	2	2	0	5
13	MSC3	0	0	0	5	4	3	2	5	2	3	3	5
14	MSC3	3	3	3	2	4	2	4	4	0	0	0	2
15	MSC3	0	0	0	0	3	1	0	0	0	0	0	0
16	MSC3	0	0	0	5	4	4	4	5	0	0	0	5
17	MSC6	0	0	0	0	4	4	4	4	0	0	2	3
18	MSC6	0	0	0	0	4	4	4	4	0	0	0	0
19	MSC6	0	0	0	5	3	3	3	5	4	3	2	5
20	MSC6	3	3	2	0	3	4	3	2	0	0	1	1
21	MSC6	0	0	0	0	3	1	0	0	1	0	0	0
22	MSC6	0	0	0	0	4	4	4	1	1	0	0	0
23	MSC6	0	0	0	0	4	4	1	0	0	0	0	0
24	MSC6	3	3	0	0	4	4	0	0	0	0	0	0
25	MSC6	0	0	0	0	4	2	2	4	0	3	0	2
26	MSC6	2	1	1	0	2	2	2	0	2	2	1	2
27	MSC6	3	3	2	0	3	2	1	1	0	0	0	0
28	MSC6	2	0	0	0	4	1	2	1	0	0	0	0
29	MSC6	2	2	2	5	4	4	4	5	0	0	0	5
30	MSC6	0	0	0	0	4	4	4	3	2	2	3	4
31	MSC6	0	0	0	5	3	3	1	5	0	0	0	5
32	MSC6	0	0	0	0	2	2	1	1	0	0	0	0
33	MSC6	0	0	0	0	2	1	0	0	0	0	0	0
34	MSC6	3	3	3	3	4	4	4	4	0	0	0	3
35	MSC6	1	0	0	0	2	2	0	0	3	2	2	2
36	MSC6	4	4	3	0	1	0	0	0	0	0	0	0
37	MSC6	3	3	0	0	2	2	0	0	0	0	0	0
38	MSC6	2	2	1	1	2	4	2	4	0	0	0	0
39	MSC6	0	0	0	0	3	3	2	1	0	0	0	0
40	MSC6	1	1	1	2	4	4	4	2	0	0	0	1
41	MSC6	3	2	3	5	4	0	4	5	0	3	2	5
42	MSC6	2	2	1	0	0	0	0	0	3	3	3	4
43	MSC6	0	0	0	5	4	3	3	5	0	0	0	5
44	MSC6	4	4	3	2	0	0	0	0	1	0	0	0
45	MSC6	0	0	0	0	4	2	1	0	0	0	0	0
46	MSC6	0	0	5	5	2	2	5	5	0	0	5	5
47	MSC6	1	0	0	0	2	2	1	4	0	0	0	0
48	MSC6	3	3	2	2	4	4	2	2	0	0	0	0
49	MSC6	4	3	2	3	0	0	0	0	0	0	0	0
50	MSC6	2	2	0	0	4	4	4	4	2	2	3	1
51	MSC6	0	0	0	0	2	1	1	0	0	0	0	0
52	MSC6	3	2	1	1	3	3	3	3	0	0	0	0
53	MSC6	3	3	2	2	4	4	4	4	0	0	0	0
54	MSC6	0	0	1	1	2	1	1	1	0	0	0	0
55	MSC6	1	1	1	0	1	0	0	0	3	3	3	2
56	MSC6	3	3	2	0	3	3	0	0	0	0	0	0
57	MSC6	2	2	1	0	4	4	3	1	0	0	0	0

Abbreviations: B, baseline; D7, stage on day 7; D14, stage on day 14; D28, stage on day 28.
0, GVHD stage 0; 1, GVHD stage 1; 2, GVHD stage 2; 3, GVHD stage 3; 4, GVHD stage 4; and 5, death.

Supplement 8. Relapse-free survival (RFS) and acute GVHD-free survival (aGVHDFS).

The median relapse-free survival (RFS) was 2 months [95% CI: 0.98-3.02] and the median acute GVHD-free survival (aGVHDFS) was 27 days [95% CI: 13.26 – 40.74]. In the SR-aGVHD subgroup of patients with stage 3-4 GI involvement, the median relapse-free survival (RFS) was 1 month [95% CI: 0.00 - 2.00], and the median acute GVHD-free survival (aGVHDFS) was 18 days [95% CI: 11.89 - 24.11].

Supplement 9. The later line treatment after MSC.

The 3rd line of treatment was initiated in 14 (25%) patients within a median of 28 days (range, 4-158) days from the previous line of treatment with ruxolitinib in one patient in the MSC3 group or with R-ECP in 7, ruxolitinib in 5, and vedolizumab in 1 patient in the MSC6 group. Two of the 14 patients (14%) to whom 3rd line treatment was initiated were alive at the last follow-up, with an estimated median survival of 2 months [95% CI 0.37-3.63]. Two patients (both in the MSC6 group) received 4th line treatment (one received ruxolitinib and another received R-ECP), and both patients died.

Supplement 10. Results of clinical studies with BM-derived MSC for the treatment of adult SR-aGVHD.

Publication	aGVHD Grade<br>Evaluation	aGVHD<br>localization	Age median<br>(range)	Supplement<br>Passage<br>Fresh vs Frozen	Dose<br>Schedule	ORR D28	OS<br>observation	OS	D28ORR<br>vs NR OS	Exclusion criteria<br>Biopsy-proven
Kabriaei et al.[12], 2009	N=31<br>Grade II – 21(68%)<br>Grade III – 7 (22%)<br>Grade IV – 3 (10%)<br>Modified Glucksberg criteria	Skin – 18 (58%)<br>GI – 18 (58%)<br>Liver – 2 (6%)	52 (34-67)	FBS<br>P5<br>Frozen	2 vs 8x10^6/kg (Prochymal)<br>Up to 2 infusions (3 day difference)	94%<br><br>CR-77%<br>PR-17%<br><br>D14 ORR-NM<br>CR – 52%<br>PR-NM	90 days	71%	NM	Treatment for aGVHD with ≥ 2mg/kg of MP for > 72 hours<br>Use of investigational agent within 30 days of randomization<br><br>Biopsy not obligatory
Von Bonin et al.[13], 2009	N=13<br>Grade III – 2 (18%)<br>Grade IV – 11 (82%)<br>Modified Glucksberg criteria	Skin – 6 (46%)<br>GI – 11 (85%)<br>Liver – 10 (77%)	58 (21-69)	PL 10%<br>P1-P2<br>Fresh and frozen	1x10^6/kg<br>1-5 (median 2) infusions	54%<br><br>CR-1 (8%)<br>PR-1 (8%)<br>MR-5 (38%)	92 days [7-261]	31%	NM	Not mentioned (NM)<br><br>Biopsy NM
Introna et al.[14], 2014	N=25<br>Grade II – 2 (8%)<br>Grade III-IV – 17 (68%)<br>2 (8%) cGVHD<br>2 (8%) overlap GVHD<br>Modified Glucksberg criteria	Skin – 13 (52%)<br>GI – 18 (72%)<br>Liver – 10 (40%)	40.5 (19-65)	PL 5%<br>Passage NM<br>Frozen	1.5x10^6/kg<br>2 Median	79%<br><br>CR-21%<br>PR-58%	1 year<br>2 year<br>oGVHD and cGVHD included	40%<br>30%	NM	Uncontrolled EBV, CMV or fungal infection<br>Poor clinical conditions with life expectancy of less than 30 days<br><br>Histology not required
Sanchez-Guijo et al[15], 2014	N=25<br>Grade II – 7 (28%)<br>Grade III – 15 (60%)<br>Grade IV – 3 (12%)<br>Modified Glucksberg criteria	Skin – 17 (68%)<br>GI – 20 (80%)<br>Liver – 7 (28%)<br><br>3-4 stage GI – 10 (40%)	NM (20-65)	PL 5%<br>P1-P3<br>Frozen	1x10^6/kg<br>4 doses on days 0,4,11,18	68%<br>**(D60 response)**<br><br>CR-44%<br>PR-24%	1 year	44%	CR28 vs PR/NR ~62 vs ~20)	Heart and pulmonary failure disease progression,<br>Uncontrolled bacterial, viral and fungal infection<br><br>Biopsy NM
Von Dalowski et al[16], 2016	N=58<br>Grade I – 1 (2%)<br>Grade II – 3 (5%)<br>Grade III – 8 (14%)<br>Grade IV – 46 (79%)<br>IBMTR criteria	Skin – 14 (41%)<br>GI – 53 (91%)<br>Liver – 25 (43%)<br><br>3-4 stage GI – 50 (86%)	55 (19-71)	PL 10%<br>P1-P2<br>Frozen	1x10^6/kg<br>1-6 infusions (median 2)	47%<br><br>CR-9%<br>PR-38%	1 year<br>2 years	19%<br>17%	CR/VGPR/ PR28 vs NR (~36 vs 18)	NM<br><br>Biopsy NM
Salmenniemi et al[17], 2017	N=18<br>Grade II – 1 (6%),	Skin – 12 (67%)<br>GI – 18 (100%)	45 (21-66)	PL 10%,<br>P1-P2	2 x10^6/kg	50%	6 months<br>12 months,	39%<br>29%	CR/VGPR/ PR28 vs	NM

	Grade III – 9 (50%),<br>Grade IV – 8 (44%)<br>Modified Glucksberg criteria	Liver – 5 (28%)		Frozen	Up to 6 infusions twice a week	CR-22%<br>VGPR-17%<br>PR-11%	24 months,	18%	NR (~52 vs 20)	Biopsy NM
Dotoli et al[20], 2017	N=30<br>All Grade III-IV (not specified by grade)<br>Grading system NM	NM adults	NM	FBS<br>P3-P5<br>Fresh	0.98 – 29.78 x 10^6/kg (median $6.81x10^6$ given each week	53%<br><br>CR-10%<br>PR-43%	1 year	19%	CR/PR28 vs NR<br>(1 year OS ~35 vs 0)	NM<br><br>Biopsy NM
Fernandez-Maqueda et al.[21], 2017	N=33 (evaluated 32)<br>Grade II – 17 (52%)<br>Grade III – 9 (27%)<br>Grade IV – 7 (21%)<br>Modified Glucksberg criteria	Skin – 22 (67%)<br>GI – 26 (79%)<br>Liver – 7 (22%)	46 (18-61)	FBS and PL<br>P1-P3<br>Frozen	1 $x10^6$/kg<br>From 1 to 16 (median 4)	84%<br><br>CR-34%<br>PR-50%	3 months	69%	CR28 vs PR/NR<br>(1-year OS 79 vs 25)	NM<br><br>Biopsy NM
Servais et al[19], 2018	N=33 (4 children)<br>Grade II – 9 (27%)<br>Grade III – 15 (45%)<br>Grade IV – 9 (27%)<br>Modified Glucksberg criteria	Skin – 17 (52%)<br>GI – 27 (82%)<br>Liver – 12 (36%)	58 (5-69)	FBS<br>P1-P3<br>Frozen	1-2 vs $3\text{-}4x10^6$/kg<br>1 or 2 infusions	41% (D30)<br><br>CR-22%<br>PR-19%	1 year	18.2%	CR/PR28 vs NR (50 vs 7.7)	Relapsing or progressing malignancy, HIV infection, Active uncontrolled infection<br><br>Biopsy NM
Bader et al.[18], 2018	N=18 (26%)<br>All patients (n=69)<br>Grade II – 3 (4%)<br>Grade III – 25 (36%)<br>Grade IV – 41 (59%)<br>**Adult grades NM**<br>Modified Gluckberg criteria	NM	45.5 (18-65)	PL 5-10%<br>P1-P2<br>Frozen	$1\text{-}2x10^6$/kg once weekly<br>1-4 doses	89%<br><br>CR-50%<br>PR-39%	6 months	61%<br>[95%CI 42-88]	NM	NM<br><br>A biopsy was only sought to rule out alternative diagnoses.
Bonig et al [24], 2019<br>[Including cohort from Bader et al., 2018]	N=31 (34%)<br>All patients (n=92)<br>Grade II – 3 (3%)<br>Grade III – 34 (37%)<br>Grade IV – 54 (59%)<br>Not specified – 1 (1%)<br>**Adult grades NM**<br>Modified Gluckberg criteria	NM	42.4 (18.4–65.6)	PL 5-10%<br>P1-P2<br>Frozen	$1\text{-}2x10^6$/kg once weekly<br>1-4 doses	Adults<br>77%<br>CR-35%<br>PR-42%<br><br>Children<br>84%<br>CR-59%<br>PR-25%	6 months	Adults<br>54%<br>[95%CI 39-76]<br><br>Children<br>69%<br>[95%CI 58-82]	NM	NM<br><br>A biopsy was only sought to rule out alternative diagnoses.

Galleu et al [22], 2019	N=60<br>NM adults<br>Grade II – 5 (8%)<br>Grade III-IV – 55 (92%)<br>Modified Gluckberg criteria	Skin, Gut, Skin + Gut – 42 (70%)<br>Other – 18 (30%)	40 (4mo-68)	PL 5%<br>P3<br>Frozen	Median 2.6 × 10^6/kg (0.6-15.6 × 10^6/kg)	53%<br>(D7 response)<br><br>CR-1 (1.6%)<br>PR-31 (51.6%)	Median 3.4 months (95% CI 0-7.8)	NA	NM<br>CR/PR D7 not reached vs non responders 0.6 months [95CI% 0.4-1.0)	NM<br><br>Biopsy not mandatory
Hinden L et al [23], 2019	N=26 (4 children)<br>Grade I-II – 5 (19%)<br>Grade III-IV – 21 (81%)<br>aGVHD – 22 (85%),<br>cGVHD -– 4 (15%)<br>Modified Gluckberg criteria	Skin – 23 (88%)<br>GI – 25 (96%)<br>Liver – 6 (23%)	NM	FBS<br>P1-P7<br>Frozen	Median –1.06 × 10^6/kg (0.59-1.8 × 10^6/kg)	50% responders (Evaluation time NM)	40 days	Responders 84.6% vs non-responders 30.8%	NM	NM<br><br>Biopsy NM
Bukauskas et al, 2024 [Author of this paper]	N=57<br>Grade III – 52 (91%)<br>Grade IV – 5 (9%)<br>Modified Glucksberg criteria	Skin – 28 (49%)<br>GI – 53 (93%)<br>Liver – 14 (25%)<br><br>3-4 stage GI – 39 (68%)	55 (19-71)	PL 5%<br>P1-P2<br>Bioreactor assisted<br>Frozen	1 x 10^6/kg<br>MSC3 – up to 3 doses<br>MSC6 – 6 doses	42%<br><br>CR-15 (26%)<br>PR-9 (16%)<br><br>D14 ORR- 39%<br>CR-7 (12%)<br>PR-15 (26%)	6 months<br>1 year<br>5 year<br>10 years	31%<br>27%<br>24%<br>24%	CR/PR28 vs NR<br>(52 vs 7)<br><br>CR/PR14 vs NR<br>(54 vs 5)	None<br><br>Mandatory biopsy

Abbreviations: NM, not mentioned; FBS, fetal bovine serum; GvHD – Graft-versus-Host Disease; PL, platelet lysate; P, passage; GI, gastrointestinal; CR, complete response; PR, partial response; ORR, overall response rate (CR + PR); aGvHD, acute GVHD; cGvHD, chronic GvHD.
